# Adaptation and evolution of the sea anemone *Alvinactis* sp. to deep‐sea hydrothermal vents: A comparison using transcriptomes

**DOI:** 10.1002/ece3.9309

**Published:** 2022-09-20

**Authors:** Zehui Xu, Zeyu Chen, Haibin Zhang

**Affiliations:** ^1^ Institute of Deep‐sea Science and Engineering, Chinese Academy of Sciences Sanya China; ^2^ University of Chinese Academy of Sciences Beijing China; ^3^ State Key Laboratory of Genetic Resources and Evolution Kunming Institute of Zoology, Chinese Academy of Sciences Kunming China

**Keywords:** adaptation, chemosynthetic ecosystem, endocytosis, hydrothermal vent, peroxisome, positively selected gene, sea anemone

## Abstract

Sea anemones are diverse and ecologically successful members of Anthozoa. They are often found in intertidal and shallow waters, although a few of them inhabit harsher living conditions, such as deep‐sea hydrothermal vents. Here, we sequenced the transcriptome of the vent sea anemone *Alvinactis* sp., which was collected from Edmond vent along the central Indian Ocean ridge at a depth of 3275 m, to explore the molecular mechanisms related to adaptation to vents. Compared with another deep‐sea anemone (*Paraphelliactis xishaensis*) and five shallow water sea anemones, a total of 117 positively selected genes and 46 significantly expanded gene families were found in *Alvinactis* sp. specifically that may be related to its vent‐specific aspect of adaptation. In addition, 127 positively selected genes and 23 significantly expanded gene families that were found in both *Alvinactis* sp. and *P. xishaensis.* Among these, vent‐specific adaptations of *Alvinactis* sp. may involve genetic alterations in peroxisome, ubiquitin‐mediated protein degradation, oxidative phosphorylation, and endocytosis, and its deep‐sea adaptation may involve changes in genetic information processing. Differentially expressed genes between *Alvinactis* sp. and the deep‐sea anemone *P. xishaensis* were enriched in a variety of pathways related to adaptation, such as energy metabolism, genetic information processing, endocytosis, and peroxisomes. Overall, we provided the first transcriptome of sea anemones that inhabit vents, which enriches our knowledge of deep‐sea hydrothermal vent adaptation and the diversity of sea anemones.

## Graphical abstract

The transcriptome of the sea anemone *Alvinactis* sp. from hydrothermal was sequenced in order to provide the probable molecular mechanisms related to adaption. Our results show that genetic changes in multiple biological processes jointly facilitate *Alvinactis* sp. to inhabit the vent environment. And this is the first transcriptome of the sea anemone living in deep‐sea vent environment.

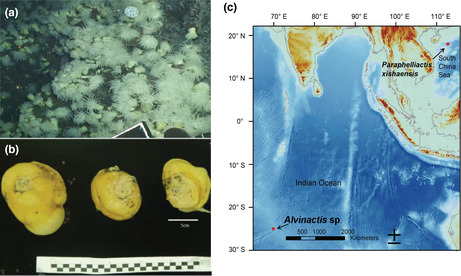

## 1 INTRODUCTION

Deep‐sea hydrothermal vents are geologically active areas that are usually located on an ocean ridge, back arc basin, or active seamount. They discharge heated, reduced fluids enriched with hydrogen sulfide (H_2_S), methane, and heavy metals (Tyler et al., 2002), which result in harsh living conditions that include highly variable and sometimes high temperature, high hydrostatic pressure, chronic hypoxia, and high concentrations of H_2_S and heavy metals. However, chemoautotrophic bacteria at vents utilize the reducible materials to synthesize nutrients and to act as primary producers to support dense macrofaunas like shrimp (Yuan et al., 2020), mussels (Sun et al., 2017; Zheng et al., 2017), or gastropods (Sun et al., 2020). Vents are oases within a relatively lifeless deep sea floor where >600 species have been reported (Van Dover et al., 2002). These endemic macrofaunas have high biomass, but low biodiversity, and biologists are curious to know how they have adapted to such an environment.

Some distinct morphological and physiological traits related to vent animals have been observed. In general, vent macrofaunas cannot tolerate high temperatures for a long time and prefer cooler temperatures (Matabos et al., 2008; Mickel & Childress, 1982a, 1982b; Smith et al., 2012), and escaping from high temperatures by moving away from the sources represents an easy and adaptive way against rapid temperature fluctuations and fluid toxicity (Bates et al., 2010). Also, enzymes in vent macrofaunas are less likely to be affected by variable temperatures compared with their shallow water relatives (Lallier & Truchot, 1997; Sanders et al., 1988; Truchot, 1992). When confronted with hypoxia, some Polychaeta exhibited greatly enlarged gill surface areas to obtain more oxygen from the hypoxic water, and their respiratory pigments (hemoglobins and hemocyanins) exhibited very high intrinsic oxygen affinities (Hourdez & Lallier, 2007).

In the presence of hydrogen sulfide (H_2_S), the defense against sulfide poisoning is to oxidize it into less toxic forms, such as thiosulfate by mitochondria (Somero et al., 1989; Vetter et al., 1987). The detoxification system can be highly efficient because no free sulfide can be found in cells (Vetter et al., 1987). Heavy metals can be sequestrated by metallothionein, phytochelatin, or ferritin to avoid their toxicity (Chen et al., 2015; Wong et al., 2015; Zapata et al., 2009). The in vivo effect of metals (like reactive oxygen species [ROS]) causes these organisms to respond by the function of detoxification of superoxide dismutase (SOD), catalase, or glutathione peroxidase (Genard et al., 2013; Marie et al., 2006).

Some molecular mechanisms for adaptation to vent environments have also been elucidated. For instance, transcriptome analysis of the shrimp, *Alvinocaris longirostris*, from the Iheya North hydrothermal vent found multiple copies of enzymes to eliminate toxic xenobiotics and various differentially expressed genes related to sulfur metabolism, detoxification, and mitochondria (Hui et al., 2018). In vent polynoid scale worms, tetra‐domain hemoglobin was found under rapid evolution in *Branchipolynoe*, and single‐domain hemoglobin was highly expressed in *Lepidonotopodium* sp. (Zhang, Sun, Chen, et al., 2017). These studies provided some insight into adaptive mechanisms for those organisms, but the adaptive mechanisms of vent sea anemones have been reported rarely.

Sea anemones are common and conspicuous species in many mid‐ocean ridge hydrothermal vent ecosystems in the Atlantic, Pacific, and Indian Oceans (Zelnio et al., 2009). Morphologically, deep sea and chemosynthetic sea anemones have some distinct features; common deep‐sea sea anemones overall are larger in form (like *Actinernus elongatus* and *Glyphoperidium bursa*) than that of shallow water anemones. Some deep‐sea clades (e.g., *Bolocera*, *Liponema*, presumably *Iosactis*) have long, deciduous tentacles and tend to have a short column (Rodríguez, 2012). Members of the deep‐sea and polar clade Actinostolina have smooth columns and often perform internal brooding to protect offspring from the extreme environment (Rodríguez et al., 2013), and the deep sea cuticulate clade in Metridioidea usually have thick columns that bear cuticles and tubercles (Rodríguez & Daly, 2010). The symbiosis of deep‐sea/vent sea anemones with chemoautotrophic bacteria is unknown, except for one species reported from vents in the Gulf of California (Goffredi et al., 2021).

The research on genomes of sea anemones has provided many surprises. The genome sequence of *Nematostella vectensis*, which was published in 2007, showed that this morphologically simple creature had a complex genomic composition like vertebrates that indicated that this eumetazoan ancestor already formed a “gene box toolkit” about 500 million years ago (Putnam et al., 2007). Another study revealed different functions of hox genes in sea anemone development compared with hox genes in vertebrates (He et al., 2018). Because sea anemones are at the sister branch of all bilaterians, it is valuable to use sea anemones to understand the origin and evolution of genes, tissues, and organs (Bosch et al., 2017). In addition, sea anemones may provide insights into related adaptations of cnidarians.

The morphology, anatomy, and phylogeny of some vent sea anemones have been described (Rodríguez et al., 2014; Rodríguez & Daly, 2010; Zelnio et al., 2009), but their genomes or transcriptomes have not been elucidated until recently. Here, we sequenced the first transcriptome of a deep‐sea vent sea anemone (*Alvinactis* sp.) collected from the Edmond vent field on the Central Indian Ridge (CIR) at a depth of 3275 m. According to in situ videos, we found white sea anemones thrived around the vents, and they comprised a large part of the vent fauna together with shrimp and gastropods. The investigation of transcriptomes of vent sea anemones will contribute to the knowledge of adaptive mechanisms of vent faunas and, therefore, increase our understanding of vent ecosystems.

## 2 MATERIALS AND METHODS

### 2.1 Sample collection, RNA extraction, and sequencing

**FIGURE 1:**
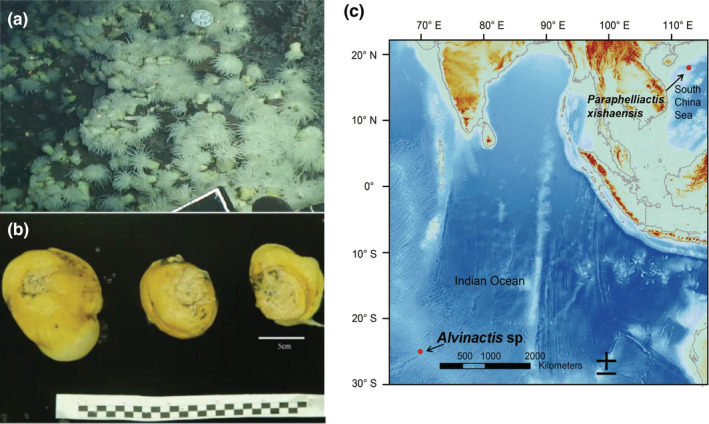
Morphology of sea anemone *Alvinactis* sp. (a) In situ photo that shows the white color of vent sea anemones near the vent. (b) Morphology of vent sea anemones on board. (c) Locations of vent and deep‐sea anemones.

Sea anemones were collected on the Edmond vent field from the CIR by the manned submersible *Shenhaiyongshi* at a depth of 3275 m (Figure 1c). After being collected, specimens were frozen immediately onboard in liquid nitrogen and then stored at −80°C before mRNA was extracted. Tissues from the body walls of three samples were sequenced separately. A TRIzol kit (Invitrogen) was used to extract total RNA, which followed the manufacturer's instructions. Sequencing libraries were constructed using a NEBNext RNA Library Prep Kit for Illumina (NEB), which followed the manufacturer's recommendations, and sequencing was done using an Illumina Hiseq 2500 for paired end sequence with a read length of 150 bp.

### 2.2 Data filtering, transcriptome assembly, and function annotation

The raw sequencing reads were first evaluated using FASTQC (www.bioinformatics.babraham.ac.uk/projects/fastqc/). Adapters and low‐quality reads were trimmed by TRIMMOMATIC version 0.39 (Bolger et al., 2014). The resultant clean reads were assembled de novo using TRINITY version 2.9.1 (Grabherr et al., 2013) with default settings. To remove redundant gene isoforms, only isoforms with the longest length were retained. Redundancy of transcripts was further removed using CD‐HIT‐EST version 4.8.1 (Fu et al., 2012) with a threshold of ≥95% sequence similarity. The completeness of the assembly was evaluated by BUSCO version 4.8.4 with the mode of transcripts (Manni et al., 2021). TRANSDECODER version 5.5.0 (http://github.com/TransDecoder/TransDecoder) was utilized to predict coding regions of all the remaining transcripts and to translate them into protein sequences with a default minimum protein length of 100 aa.

All predicted protein sequences were aligned to the NCBI non‐redundant database (NR) and the Swiss‐Prot database using BLASTp (E‐value <1 × e^−7^) to produce annotation results. EggNOG‐mapper (Huerta‐Cepas et al., 2019) was used to perform genome annotation and gene ontology annotation. The KEGG (Kyoto Encyclopedia of Genes and Genomes) Automatic Annotation Server (Moriya et al., 2007) was used with the bidirectional BLAST method to identify pathway information. Gene function enrichment was conducted using KABOS version 3.0 (Xie et al., 2011) with annotations for *Nematostella vectensis* (sea anemone) as background.

### 2.3 Species identification and public data selection

Three mitochondrial genes (12S, 16S, and cox3) and two nuclear genes (18S and 28S) were used to identify the vent sea anemone. The five genes were extracted from the assembled transcriptome and blasted against the online NCBI nt database. Based on the morphology and the blast results (Table S1), we identified the vent sample as *Alvinactis* sp. A total of 18 publicly accessible transcriptome or genome datasets were used for comparative analysis, which included 16 sea anemones (Table S2), one Corallimorpharia, and one Scleractinia. Among them was *Paraphelliactis xishaensis*, which is a deep‐sea anemone found in the Xisha Trough in the South China Sea at a depth of 3230 m. The Corallimorpharia was *Corynactis australis*, and the Scleractinia was *Acropora digitifera*. Completeness of assemble transcripts or genes annotated from genome was confirmed by BUSCO version 4.8.4 (Manni et al., 2021) with a metazoan database (Table S1).

### 2.4 Identification of orthologs and phylogenetic analysis

**FIGURE 2:**
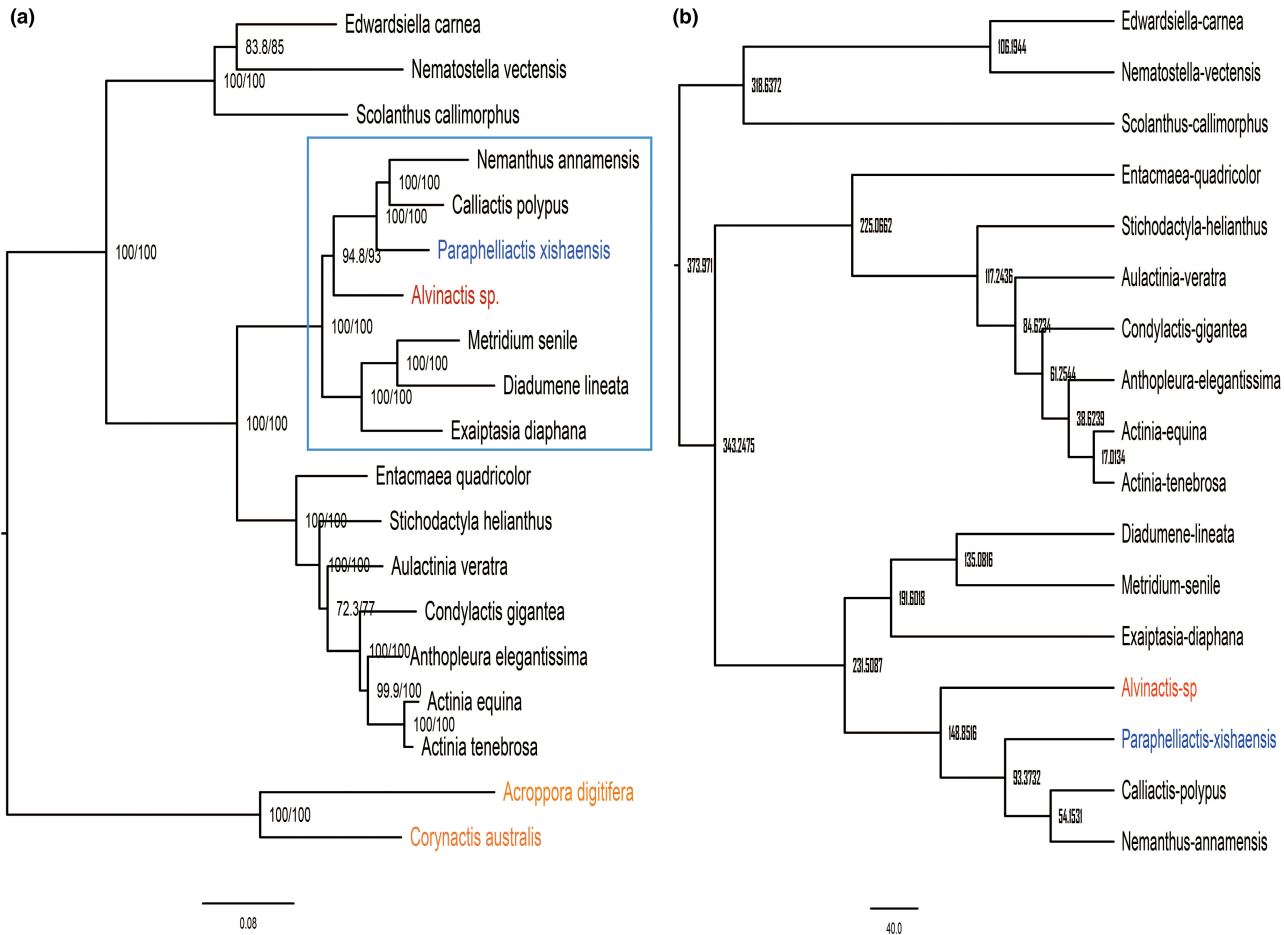
Phylogenetic relationship among sea anemones. (a) Maximum‐likelihood phylogenetic tree of sea anemones. Numbers above branches indicate support value, number in left of the slash indicates bootstrap value, and the number on the right indicates SH‐like approximate likelihood ratio. Red label represents vent sea anemone, blue represents deep‐sea anemone, orange represents outgroup, and black represents shallow water sea anemone. Rectangle with blue frame contains phylogenetically closely related species to *Alvinactis* sp. (b) the divergence time of sea anemones.

OrthoFinder version 2.3.11 (Emms & Kelly, 2015) was used to identify the orthologs among sea anemones with diamond (Buchfink et al., 2015) chosen for protein alignment. Single copy orthologs were aligned by MAFFT (Katoh et al., 2009), and conserved regions were contracted by Gblocks (Talavera & Castresana, 2007). Then, they were concatenated and used to construct a phylogenetic tree using IQtree version 1.6.12 (Nguyen et al., 2015) with a bootstrap of 1000 and SH‐like approximate likelihood ratio test with the parameter “iqtree2 ‐s align.phy –alrt 1000 ‐b 1000 ‐T AUTO.” Corallimorpharia and Scleractinia were designated as outgroups. Based on the phylogenetic relationship revealed by the above process, six sea anemones (*Paraphelliactis xishaensis*, *Calliactis polypus*, *Nemanthus annamensis*, *Exaiptasia diaphana*, *Metridium senile*, and *Diadumene lineata*) together with our vent sample were used to perform the following positive selection and gene family expansion/contraction analysis (Figure 2a). The divergence time was estimated by MCMCtree (Yang, 2007) with time calibrations based on the estimated time from TIMETREE (timetree.org): Edwardsiidae‐Actinioidae‐Aiptasiidae 338 MYA (dos Reis et al., 2015; Schwentner & Bosch, 2015) and Actiniidae‐Aiptasiidae 334 MYA (Schwentner & Bosch, 2015).

### 2.5 Positive selection analysis

Our vent sample *Alvinactis* sp. and the six sea anemones listed above were used to get orthogroups by Orthofinder as described above. Also, the proteins of all seven species were put into one file, and we performed an all vs all blast. Orthogroups with at least one ortholog in each sea anemone were selected, and then, we picked out the longest ortholog of *P. xishaensis* and the ortholog of the other six sea anemones that had the highest identity to *P. xishaensis*. Codon alignment was conducted by ParaAT (Zhang et al., 2012) for PAML with parameter “ParaAT.pl ‐a all.pep ‐n all.cds ‐h single_copy_orthogroups ‐p proc ‐f paml ‐g,” and ‐g indicated no gap. Identification of positively selected genes was performed using Codeml in PAML version 4.9 (Yang, 2007). We ran Codeml two times. In the first run, we set the vent sea anemone *Alvinactis* sp. as foreground to identify genes that were particularly related to vent adaption, and, in the second run, we set both the vent sea anemone *Alvinactis* sp. and the deep‐sea anemone *P. xishaensis* as foreground to identify genes related to deep‐sea adaption because these two species were both living in deep‐sea environments. Codeml was performed with an optimized branch‐site model combined with Bayesian Empirical Bayes (BEB) methods. This model compared two hypotheses where the null hypothesis assumed that ω in all branches was <1, and the alternative hypothesis assumed that ω in foreground branches was >1. Chi‐square tests were applied to check whether two times the difference between the maximum likelihood value (lnL) of the alternative hypothesis (ω = 1 and Fix ω = 0) and the null hypothesis (Fix ω = 1) were two times greater than the threshold at the given free ratio and *p*‐value. The function p.adjust in R‐3.5.0 was used to get the adjusted *p*‐value.

### 2.6 Gene family evolution

**FIGURE 3:**
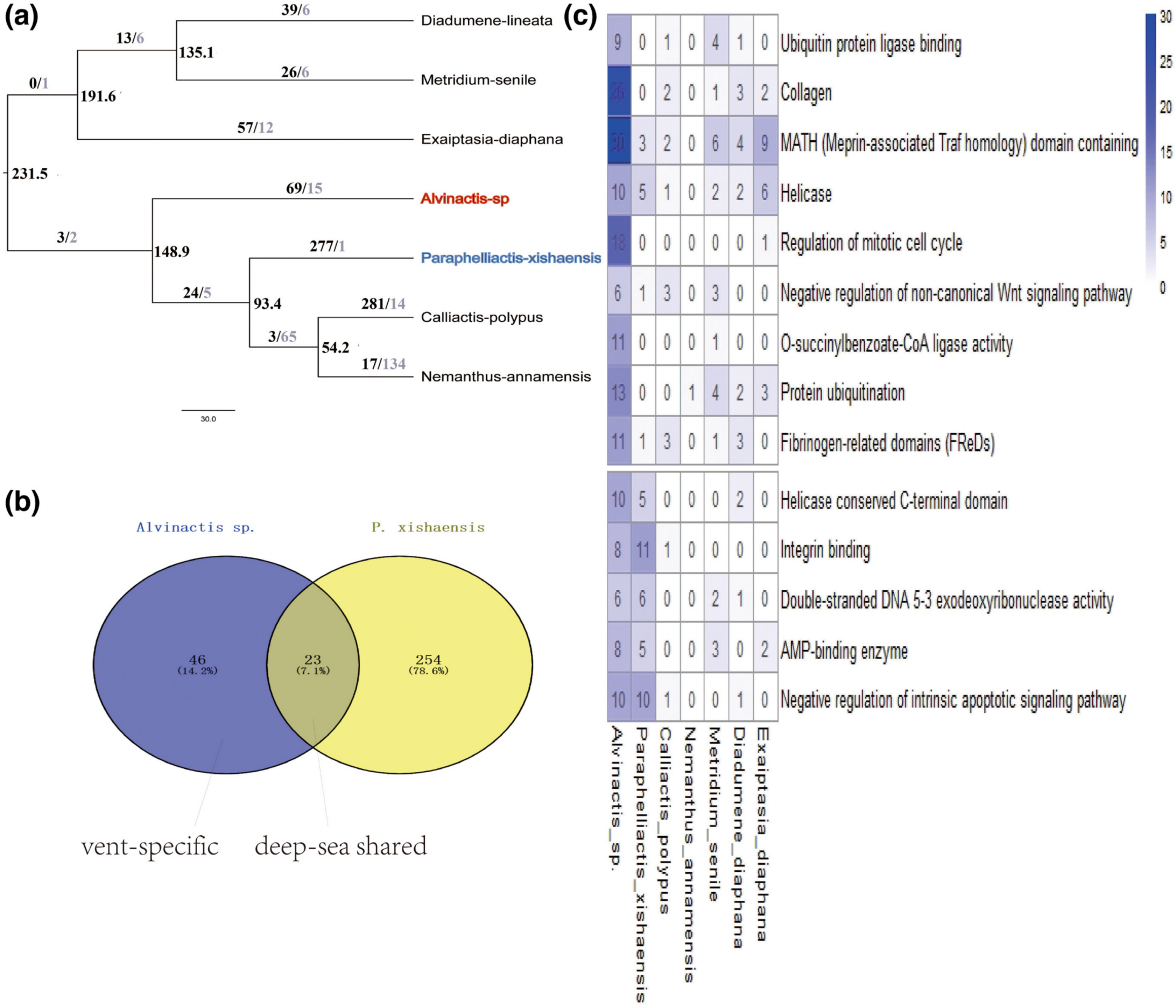
Significantly expanded gene families in *Alvinactis* sp. (a) The expansion and contraction of gene families among sea anemones, black colored number indicates expansion and gray colored number indicates contraction. (b) The intersection of expanded gene families between vent and deep‐sea anemone. (c) Heatmap of the expanded gene families. *X*‐axis indicates species used for comparison, which are roughly ordered by phylogenetic distance to vent sea anemone; *y*‐axis indicates annotation/function of gene families. Color correlates with gene number.

The expanded and contracted gene families on each branch were identified by CAFE5 (Mendes et al., 2020) among seven sea anemones used in above positive selection analysis; gene counts of each orthogroup and an ultrametric tree calculated by MCMCtree were used as input for CAFE5. By comparing the expanded gene families of the vent sea anemone *Alvinactis* sp. and the deep‐sea anemone *P. xishaensis*, gene families uniquely found in *Alvinactis* sp., but not in *P. xishaensis*, were defined as vent‐specific expanded gene families. Gene families both found in the two anemones were defined as deep‐sea shared expanded gene families. A Venn diagram was used to show the relationships of expanded gene families between *Alvinactis* sp. and *P. xishaensis* (Figure 3b).

### 2.7 Differential gene expression

**FIGURE 4:**
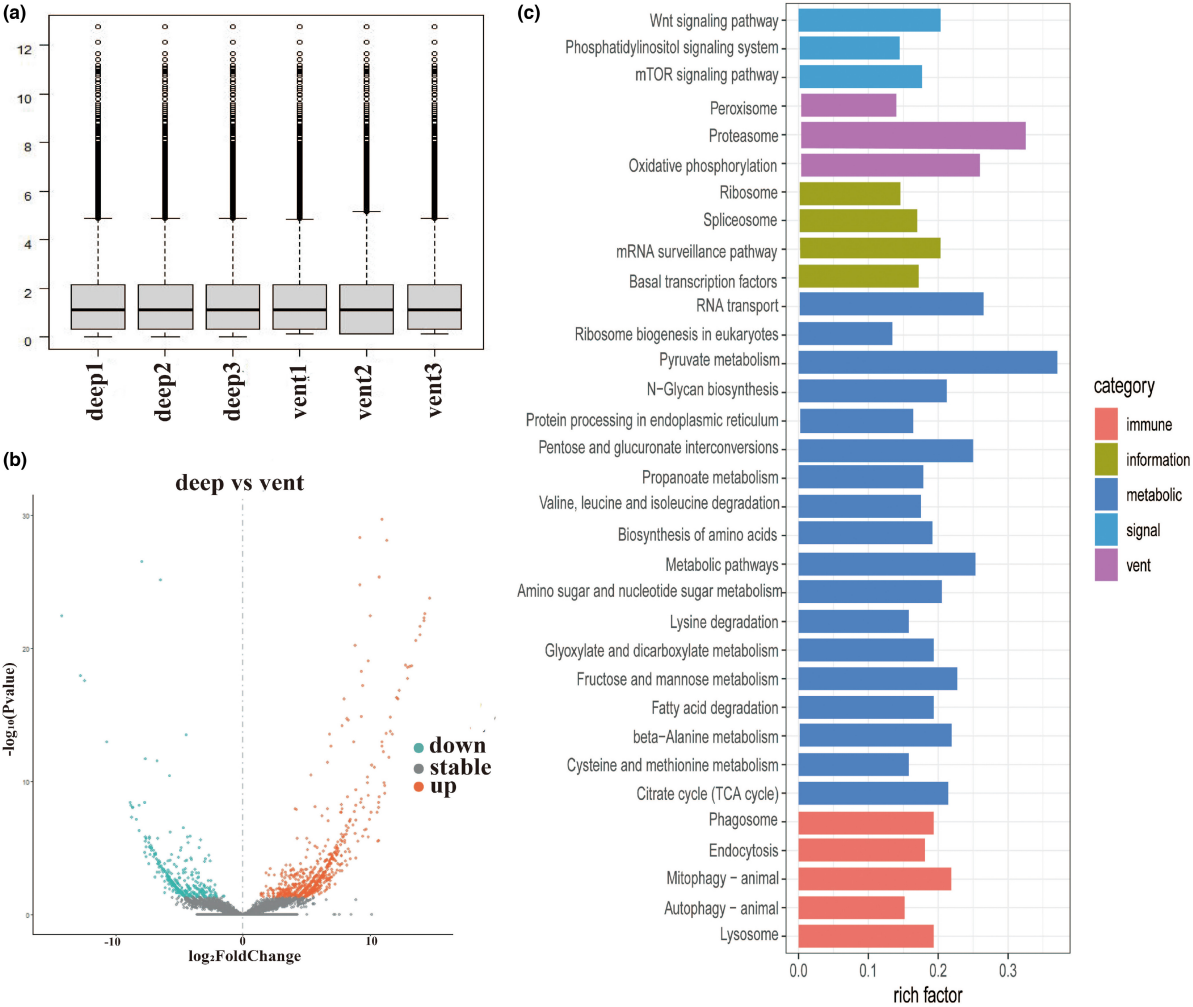
Comparisons of transcriptomes between vent and deep‐sea anemones. (a) Expression levels after log_2_ transformation and normalization. (b) Differentially expressed orthologs between deep‐sea and vent sea anemone. (c) Functional enrichment of differentially expressed orthologs. The *x*‐axis shows the enriched ratio between input and background.

In vent or deep‐sea anemones, three replicates of transcriptomes from body walls were chosen. Relative levels of gene expression were calculated by mapping clean reads to their own species‐specific assembled transcriptome using align_and_estimate_abundance.pl in Trinity, and abundance_estimates_to_matrix.pl was used to get a matrix of normalized expression values with the TMM method implemented in edgeR (Robinson et al., 2010). Transcript abundances were estimated as TPM (Transcripts Per Million reads). Then, we used the orthologs of vent and deep‐sea anemones from the above positive selection analysis to compare their differential ortholog expression. Some normalization steps were added further for comparison of transcriptomes between different species (Gan et al., 2020; Zancolli et al., 2022). First, we minimized the effects of technical artifacts by quantile normalization on log_2_‐transformed TPM values, to which a pseudo counts of 1 was added to prevent log2(0) scores. Then, we used ComBat function in the sva R package (Leek et al., 2012) to remove the batch effects caused by using multiple species (Figure 4a). EdgeR was used for differential expression with the TPM values compared (Robinson et al., 2010). A *q*‐value of <0.05 and a fold change of >2 was considered significant. The DEGs were annotated functionally, and enrichment was analyzed using KOBAS (Bu et al., 2021) and DAVID (Sherman et al., 2022).

## 3 RESULTS

### 3.1 De novo assembly and annotation of the vent sea anemone transcriptome

A total of 85,574,359 raw reads with a length of 150 bp of *Alvinactis* sp. were generated. After trimming reads with low quality and adapters, 82,698,960 (96.64%) reads remained. Transcriptome assembly got 127,084 unigenes with an N50 length of 1590 bp. The unigenes hit 90.4% of the complete orthologs in the BUSCO database, which included 86.1% single‐copy and 4.3% duplicated. Among these unigenes, 55.1% of them were annotated by at least one database (Table 1).

**TABLE 1:** Summary of assembly and annotation for the deep sea, hydrothermal vent sea anemone *Alvinactis* sp*.*

	*Alvinactis* sp.
Trinity assembly	
Number of contigs	249,865
Average contig length	884.47
Number of unigenes	127,084
N50 length of unigenes	1590
Average length of unigenes	691.05
Annotation	
Annotated proteins	32,870
PFAM	19,603 (49.1%)
KEGG	15,826 (48.1%)
GO	13,214 (40.2%)
NR	16,234 (49.4%)
SWISS‐Prot	16,568 (50.4%)
At least one database	28,078 (55.1%)

Abbreviations: GO, Gene ontology; KEGG, Kyoto Encyclopedia of Genes and Genomes; NR, non‐redundant database; PFAM, Protein family.

### 3.2 Species identification

With the blast results (Table S1) from the NCBI BLAST online database, molecular markers 12S, 16S, and COX3 indicated our vent sea anemone was *Alvinactis chessi* with an identity >99.3%, 18S indicated *Sagartiogeton erythraios* with an identity of 97.66%, and *Kadosactis antartica* with an identity of 97.75%. 28S indicated *Anthosactis pearseae* with an identity of 98.68% and *Heteranthus verruculatus* NY411 with an identity of 97.45%. Although 18S and 28S did not indicate *Alvinactis chessi* with the highest identity score, we still identified our vent sea anemone as *Alvinactis* sp. due to its morphological similarity to *Alvinactis* sp. found in Pacific Ocean (Figure 1).

### 3.3 Phylogenetic tree and divergence time

A phylogenetic tree was built based on the alignment of 75,298 amino acids from 86 single‐copy orthogroups with the Scleractinia (*Acropora digitifera*) and Corallimorpharia (*Corynactis australis*) as outgroups (Figure 2a). This phylogenetic relationship among sea anemones was comparable to the work of Rodríguez et al. (2014). To make sure that most of the genetic differences were from different living conditions rather than phylogenetic distance, we chose these two deep‐sea anemones and another five shallow water anemones that were closely related phylogenetically (Figure 2a) to conduct the following positive selection and gene family expansion/contraction analysis. Based on the calibration time shown in the TIMETREE (timetree.org), we checked the divergence time among these sea anemones. *Alvinnactis* sp. diverged from other sea anemones around 148.8 MYA (Cretaceous Period), and the deep‐sea anemone *P. xishaensis* diverged from other sea anemones around 93.4 MYA (Figure 2b, Table S8).

### 3.4 Positively selected genes

Environmental forces that have acted on species can be reflected by fitted positively selected genes (Nielsen, 2005). These genes encode proteins with some novel functions to acclimate to any environmental change. We used Codeml to test for signatures of positive selection. When we designated the vent sea anemone, *Alvinactis* sp., as the foreground branch, 117 (vent‐specific) positively selected genes (PSGs) were identified from 9649 orthogroups (Table S3). When we designated the deep‐sea anemones (*Alvinactis* sp. and *P. xishaensis)* as foreground branches, 127 (deep‐related) PSGs were identified (Table S4). Ten PSGs were shared using the above two tests. Here, we regarded vent‐specific PSGs as more related to vent adaptation. Functions of the vent‐specific PSGs (Table 2) were mainly enriched in pathways related to Peroxisome (ACOX1 and EPHX2), ubiquitin‐mediated proteolysis (APC8, CDC20, CUL2, parkin, FBXW7, and BIRC2_3), endocytosis (RAB7A, EPS15, and CHMP7), apoptosis (BAK1, BIRC2_3, and PARAP1), and metabolism. The deep‐sea related PSGs (Table 2) were enriched in pathways related to protein processing in the endoplasmic reticulum (EIF2S1, SEC62, and UBE2G1), ubiquitin‐mediated proteolysis (BTRC, UBE2C, and UBC7), autophagy (EIF2S1, PRKAA, RAB1A, and RUBCN), and Wnt (WNT6, BTRC, and PPP3C) signaling pathways.

**TABLE 2:** Functional enrichment of vent‐specific and deep‐sea related positively selected genes in vent sea anemone (*Alvinactis* sp.)

	Input number	Background number	*p*‐Value	Corrected *p*‐value
Term (vent‐specific)				
Ubiquitin mediated proteolysis	5	92	0	.0021
Apoptosis – multiple species	2	15	.0024	.0401
mRNA surveillance pathway	2	62	.0316	.1737
Peroxisome	2	66	.0353	.1737
Ubiquinone and other terpenoid‐quinone biosynthesis	1	8	.0384	.1737
Metabolic pathways	8	908	.0460	.1737
Autophagy – animal	2	79	.0484	.1737
Endocytosis	3	116	.0492	.1823
Term (deep‐sea related)				
Autophagy – animal	3	79	.0076	.1250
Ubiquitin mediated proteolysis	3	92	.0114	.1250
Mitophagy – animal	2	32	.0120	.1250
Protein processing in endoplasmic reticulum	3	105	.0161	.1250
Cysteine and methionine metabolism	2	38	.0164	.1250
Valine, leucine, and isoleucine biosynthesis	1	5	.0290	.1839
Wnt signaling pathway	2	59	.0361	.1961
Notch signaling pathway	1	9	.04794	.2163

### 3.5 Expanded gene families

Gene duplication is one of the main genomic resources for adaptive evolution, and duplicated genes can be expressed together to enhance the original function or to evolve new functions. Among the 23,655 gene families of the seven sea anemones used above, 69 gene families were expanded in *Alvinactis* sp., and 277 gene families were expanded in *P. xishaensis* (Figure 3a). Here, the last common ancestor (the nearest internal node) of *Alvinactis* sp. also represented the common ancestor of *P. xishaensis* and the two shallow water sea anemones *Calliactis polypus* and *Nemanthus annamensis* (Figure 3a); we did not use the expansion in the ancestor node to represent the expanded gene families shared by vent and deep‐sea anemones. By comparing the expanded gene families of *Alvinactis* sp. and *P. xishaensis* (Figure 3b), we found 46 gene families (12 of them can be annotated except transposons) that were expanded specifically (vent‐specific) in *Alvinactis* sp. (Table S5). Twenty‐three expanded gene families (five of them can be annotated except transposons) were shared (deep‐sea shared) by *Alvinactis* sp. and *P. xishaensis* (Table S6), and the shared expanded gene families may relate to the deep‐sea related part of adaptation by vent sea anemones.

None of the 69 expanded gene families in *Alvinactis* sp., 46 vent‐specific expanded gene families or 23 deep‐shared gene families could be enriched in any pathways. These vent‐specific expanded gene families contained ACSF2 that was related to o‐succinylbenzoate‐CoA ligase activity and genes only with annotated function like ubiquitin protein ligase binding, hemicentin, collagen, MATH domain containing, helicase, regulation of mitotic cell cycle, protein ubiquitination, and Fibrinogen‐related domains. The deep‐sea shared expanded gene families contained EDIL3 related to integrin binding, ATAD5 related to negative regulation of the intrinsic apoptotic signaling pathway in response to DNA damage, and genes only with annotated functions like double‐stranded DNA 5′‐3′ exodeoxyribonuclease activity and AMP‐binding enzyme (Figure 3c).

### 3.6 Differentially expressed orthologs among vent and shallow‐water sea anemones

After the normalization steps (Figure 4a), there were 1197 orthologs differentially expressed (Figure 4b) between *Alvinactis* sp. and *P. xishaensis*. A total of 374 orthologs were upregulated in *Alvinactis* sp., and 823 orthologs were downregulated in it. We combined the upregulated and downregulated expression orthologs as altered expressed orthologs to learn their functions related to vent adaptation. Functions of these orthologs were enriched in a variety of pathways (Table S7, Figure 4c), such as protein synthesis (protein processing in endoplasmic reticulum and ribosome biogenesis in eukaryotes, proteasome), metabolic pathways (pyruvate metabolism, fructose and mannose metabolism, beta‐alanine metabolism, propanoate metabolism, fatty acid degradation, lysine degradation, and N‐glycan biosynthesis), energy metabolism (oxidative phosphorylation and TCA cycle), genetic information processing (mRNA surveillance pathway, RNA transport, and spliceosome), and immune‐related process (endocytosis, autophagy, mitophagy), and peroxisomes.

## 4 DISCUSSION

Although sea anemones are considered among the most ecologically successful cnidarians at all latitudes and depths of the ocean, it is still amazing to see *Alvinactis* sp. live so abundantly around the harsh environment of deep‐sea vents. The systematic relationship among sea anemones in our study was consistent with previous research (Rodríguez et al., 2014). Adding the deep‐sea anemone *P. xishaensis* to our analysis helped track genetic changes related to deep‐sea adaptation in the sea anemone *Alvinactis* sp. Also, the relatively large number of shallow water sea anemones used in our analysis makes the results more solid. Genetic changes related to *Alvinactis* sp. can be categorized mainly into vent‐specific adaptation and deep‐sea adaptation.

### 4.1 Changes related to vent‐specific adaptation

Hydrothermal vent fluids are commonly enriched in metals and metal sulfides, and metal exposure can generate an imbalance in ROS. ROS can in turn cause lipid peroxidation, protein modifications, and DNA damage (Lushchak, 2011). Metals also influence many metabolic processes directly or indirectly, including metabolism, membrane transport, and protein synthesis, and may act on DNA by interference with genetic control and repair mechanisms (Company et al., 2006; Hartwig, 1994; Hassoun & Stohs, 1996; Yamada et al., 1993). The vent‐specific PSGs EPHX2 and ACOX (Table S3) are related to peroxisome. EPHX2 participates in epoxide metabolism, which belongs to the antioxidant system, and it can detoxify xenobiotic compounds (Marowsky et al., 2017). ACOX is involved in the first step of peroxisomal β‐oxidation by catalyzing the desaturation of fatty acid‐derived side chains (Zhang et al., 2015). Moreover, DEGs were also enriched in the peroxisome pathway (Table S7, Figure 4c), which suggested that they play a role in interacting with ROS to reduce oxidative stress (Company et al., 2006; Marie et al., 2006). PSGs in the ubiquitin‐mediated proteolysis pathway may be a response to protein modification and misfolding caused by ROS; these changes may result in degradation of mis‐folded proteins or be less sensitive to protein modifications. Six PSGs that were involved (F‐BOX, cul2, cdc20, apc8, Parkin, and IAPs) belonged to the RING finger type E3 ligase (Table S3); genes (only annotated their function) related to ubiquitin protein ligase binding and protein ubiquitination were also expanded (Figure 3c). In addition, DEGs related to metabolic pathways, protein synthesis, and DNA‐related genetic information processing (Figure 4c) may also be a response and adaptation to exposure to metals near vents.

Constant exposure to H_2_S can limit the ability of organism to survive and to reproduce. H_2_S can inhibit cytochrome c oxidase (COX) in the mitochondrial respiratory chain, which interferes with ATP production (Cooper & Brown, 2008). The first line of defense against sulfide poisoning is to oxidize it to a less toxic form, such as thiosulfate (Vetter et al., 1987). The pathway for oxidative phosphorylation suggests a nexus of H_2_S toxicity and detoxification. Specifically, sulfide oxidation to thiosulfate is mediated by sulfide quinone reductase (SQR), sulfur dioxygenase, and sulfur transferase. Two vent‐specific genes for sulfur transferase (Table S3) were positively selected (i.e., GAL3ST1, which encodes galactosylceramide sulfotransferase and HS3ST3B1, which encodes heparan sulfate glucosamine 3‐O‐sulfotransferase 3B1). Genes in oxidative phosphorylation were also differentially expressed.

Endocytosis is another pathway enriched by vent‐specific PSGs (Table 2) and DEGs (Table S7). Numerous deep‐sea macrofauna that live near vents form symbiotic associations with chemosynthetic bacteria, but this association has not been documented in sea anemones previously. For example, the sea anemone *Ostiactis pearseae* that inhabits vents in the Gulf of California was confirmed to have chemosynthetic symbiosis (Goffredi et al., 2021). Endocytosis is reported to be related to the acquisition of endosymbionts. Moreover, widespread environmental viruses and bacteria around vents can take advantage of the endocytosis machinery to penetrate cytosol and use cells of host organisms as protected sites for replication (Cossart & Helenius, 2014). Three vent‐specific genes showed signals of positive selection (Table S3) in endocytosis. Eps15 is an endocytic adaptor protein involved in membrane morphology and is required for early stages of clathrin‐mediated endocytosis (Gucwa & Brown, 2014; Wang et al., 2016). Rab7 is an important regulator of late endocytic membrane traffic (Feng et al., 1995). CHMP7 is related to endosomal sorting (Horii et al., 2006). The positively selected gene Bak (Table S3) is among the core regulators of apoptosis, which can mediate the permeabilization of the outer membrane of mitochondria (Peña‐Blanco & García‐Sáez, 2018) and can regulate the homeostasis of the host‐symbiont system. Therefore, the alteration in endocytosis pathway may be an adaptation to the microbe‐rich vent environment.

### 4.2 Changes related to the deep‐sea environment

High hydrostatic pressure can cause DNA damage and form unfavorable structures of nucleic acids and proteins, which may hinder the processing of genetic information (Bourns et al., 1988). In this study, genes related to helicase were expanded in vent sea anemone (Table S6, Figure 3c). Helicase can unwind the double‐stranded nucleic acid and function in DNA modification processing, including DNA replication, DNA repair, recombination, transcription, and translation (Jankowsky & Fairman, 2007). The expansion of helicase gene family may help sustain the normal genetic information processing in *Alvinactis* sp. Genes related to integrin were also expanded (Table S6, Figure 3c), integrins are a large family of transmembrane receptors that connect cells to the extracellular matrix and help cells receive environmental information (Wu et al., 2017).

## 5 CONCLUSION

In summary, we reported the first transcriptome of a hydrothermal vent sea anemone, and we identified positively selected genes, expanded gene families, and differentially expressed genes in *Alvinactis* sp., which provides insights into the molecular adaptations to the vent environment. Due to changes in nucleotide sequences and expression levels, some distinct genes and pathways like peroxisome, ubiquitin‐mediated proteolysis, oxidative phosphorylation, and genetic information processing have been altered in *Alvinactis* sp. All these changes may help *Alvinactis* sp. to gain new molecular functions and to develop an efficient regulatory network to adapt to this harsh environment. However, our results are mainly based on the functional interpretation of homolog genes, and the specific role of these changes needs to be confirmed and investigated further. This work provides genomic resources and clues for understanding the genetic adaptations of sea anemones around hydrothermal vents.

## AUTHOR CONTRIBUTIONS

**Zehui Xu:** Formal analysis (lead); writing – original draft (lead); writing – review and editing (equal). **Zeyu Chen:** Formal analysis (supporting); methodology (supporting). **Haibin Zhang:** Funding acquisition (lead); investigation (equal); project administration (lead); supervision (equal); writing – review and editing (equal).

## CONFLICT OF INTEREST

The authors declare that that they have no competing interests.

## Supporting information


Tables S1–S8

## Data Availability

The raw transcriptomic data were deposited in the NCBI database with accession numbers SRR14268558‐560 under BioProject PRJNA722768.
