# Supplementary material for: Adaptation and evolution of the sea anemone Alvinactis sp. to deep‐sea hydrothermal vents: A comparison using transcriptomes
Source: Ecol Evol. 2022 Sep 20;12(9):e9309. doi: 10.1002/ece3.9309 (PMC9486505; doi:10.1002/ece3.9309)
Supplement: Supplementary file 1 — Tables S1–S8 [file ECE3-12-e9309-s001.docx]

**Table S1.** The identity of genetic markers (12s, 16s, 18s, 28s, cox3) blasted to BLAST NT database

| Scientific Name | E value | Per. ident | Acc. Len | Accession |
| --- | --- | --- | --- | --- |
| Alvinactis chessi_12s | 0 | 99.63 | 852 | GU473278.1 |
| Antipodactis awii_12s | 0 | 99.15 | 844 | GU473271.1 |
| Metridium senile_12s | 0 | 98.55 | 17444 | HG423143.1 |
| Alvinactis chessi_16s | 0 | 99.31 | 482 | GU473296.1 |
| Cyananthea hourdezi_16s | 0 | 99.31 | 482 | GU473293.1 |
| Kadosactis antarctica)16s | 0 | 99.31 | 480 | EU190782.1 |
| Jasonactis erythraios_18s | 0 | 97.66 | 1751 | GU473305.1 |
| Kadosactis santarctica_18s | 0 | 97.75 | 1735 | EU190865.1 |
| Metridium senile_18s | 0 | 97.24 | 1768 | KJ483035.1 |
| Ostiactis_pearseae_28s | 1.00E-108 | 98.68 | 1004 | EU190841.1 |
| Heteranthus_verruculatus_28s | 2.00E-107 | 97.45 | 261 | MW158883.1 |
| Heteranthus _verruculatus_28s | 5.00E-103 | 97.36 | 234 | MW158885.1 |
| Alvinactis chessi_cox3 | 0 | 99.85 | 706 | GU473352.1 |
| Kadosactis antarctica_cox3 | 0 | 98.36 | 715 | FJ489504.1 |
| Jasonactis erythraios_cox3 | 0 | 98.36 | 706 | GU473339.1 |

**Table S2.** BUSCO evaluation, Environmental condition, raw data accession and reference of the transcriptome assembly

|  | ***Alvinactis sp.*** | ***Calliactis***  ***polypus*** | ***Metridium***  ***senile*** | ***Nemanthus***  ***annamensis*** | ***Stichodactyla***  ***helianthus*** | ***Anthopleura***  ***buddemeieri*** | |
| --- | --- | --- | --- | --- | --- | --- | --- |
| **Complete BUSCOs** | 98.1% | 96.1% | 93.5% | 97.0% | 90.4% | | 92% |
| **Complete and single-copy BUSCOs** | 38.5% | 66.3% | 92.9% | 69.3% | 49.6% | 49.2% | |
| **Complete and duplicated BUSCOs** | 59.6% | 29.7% | 0.6% | 27.7% | 40.8% | 42.8% | |
| **Environmental conditions** | Kairei vent, 3275m from surafce | Shallow water, aims to study regeneration | Shallow water | Shallow water, to know immunome | Shallow water, to study venom and neuron | Shallow water, to know immunome | |
| **Raw data** | SRR1468558-560 | SRR4425993-6004 | SRR6480802 | SRR3228732 | SRR7126073 | SRR3193961, SRR3205971 | |
| **Reference** | This study | Stewart et al. (2017) | None (by University of Vienna)  PRJNA430035 | Van et al. (2016) | [Rivera-de-Torre](https://www.researchgate.net/profile/Esperanza-Rivera-De-Torre) et al. (2018) | Van et al. (2016) | |

**Table S2.** BUSCO evaluation, Environmental condition, raw data accession and reference of the transcriptome assembly-Continued

|  | ***Aulactinia***  ***veratra*** | ***Condylactis***  ***gigantea*** | ***Actinia***  ***equina*** | ***Diadumene***  ***lineata*** | ***Entacmaea***  ***quadricolor*** | ***Nematostella***  ***vectensis*** | |
| --- | --- | --- | --- | --- | --- | --- | --- |
| **Complete BUSCOs** | 91.5% | 97.2% | 96.7% | 93.6% | 96.4% | | 97.6% |
| **Complete and single-copy BUSCOs** | 74.8% | 55.8% | 61.9% | 47.2% | 54.7% | 51.8% | |
| **Complete and duplicated BUSCOs** | 16.7% | 41.4% | 34.8% | 46.4% | 41.7% | 45.8% | |
| **Environmental conditions** | Shallow water, to know immunome | Shallow water, to study venom and neuron | Shallow water | Shallow water | Shallow water | Shallow | |
| **Raw data** | SRR3205707-8 | ERR2045160-5 | Assembled genome and annotated by ourselves | SRR8297741 | ERR2045160-171 | Genome sequence data | |
| **Reference** | Van et al. (2016) | None (by University of Oklahoma)  PRJEB21970 | Wilding et al. (2020) | None (by QUT)  PRJNA507679 | None (by University of Oklahoma)  PRJEB21970 | Putnan et al. (2007) | |

**Table S2.** BUSCO evaluation, Environmental condition, raw data accession and reference of the transcriptome assembly-Continued

|  | ***Paraphelliactis xishaensis*** | ***Actinia***  ***tenebrosa*** | ***Anthopleura***  ***elegantissima*** | ***Exaiptasia***  ***diaphana*** | ***Edwardsiella***  ***carnea*** | ***Scolanthus***  ***callimorphus*** | |
| --- | --- | --- | --- | --- | --- | --- | --- |
| **Complete BUSCOs** | 85.6% | 96.4% | 92.0% | 93.7% | 95.1% | | 97.4% |
| **Complete and single-copy BUSCOs** | 84.8% | 84.9% | 49.2% | 82.9% | 38.1% | 32.5% | |
| **Complete and duplicated BUSCOs** | 0.8% | 11.5% | 42.8% | 10.8% | 57% | 64.9% | |
| **Environmental conditions** | South China sea deep-sea | Shallow water | Shallow water, to study venom | Shallow water, to study coral symbiosis | Shallow water, to study parasitic stage within comb jellies | Shallow water | |
| **Raw data** | Genome sequence data | SRR2437124  SRR4677512-8 | SRR1646677  SRR1645256 | Genome sequence data | SRR5931773-4  SRR6480800-1 | TSA data  SRR6480803 | |
| **Reference** | Feng et al. (2021) | Surm et al. (2019) | Macrander et al. (2015) | Baumgarten et al. (2015) | Dnyansagar et al. (2018) | None (by University of Vienna)  PRJNA430035 | |

**Table S3.** List of positively selected genes from vent sea anemone as fore branch. Ortholog, Gene symbol, p-value, adjusted p-value, Annotation is listed.

| ortholog | gene symbol | p-value | adjusted p-value | annotation |
| --- | --- | --- | --- | --- |
| diadumTRINITY_DN15879_c1_g2_i1.p1 | FLCN | 0.000003083 | 0.000234308 | negative regulation of cell proliferation involved in kidney  development |
| diadumTRINITY_DN22542_c0_g2_i14.p1 | GDAP2 | 0.000112931 | 0.007679308 | Ganglioside induced differentiation associated protein 2 |
| exaipTRINITY_DN20743_c0_g1_i1.p1 | COPZ1 | 0.000786849 | 0.049571487 | intra-Golgi vesicle-mediated transport |
| exaipTRINITY_DN25524_c0_g1_i1.p1 |  | 0.000032753 | 0.002390969 |  |
| exaipTRINITY_DN90456_c0_g1_i1.p1 |  | 0.000043528 | 0.003090488 |  |
| metriTRINITY_DN3807_c0_g1_i3.p1 | NAB1 | 0.000037113 | 0.002672136 | negative regulation of transcription, DNA-templated |
| nemantTRINITY_DN17517_c0_g1_i1.p1 | NDUFV1 | 0.000000417 | 3.21E-05 | FMN binding |
| nemantTRINITY_DN6140_c0_g1_i3.p1 |  | 0.000073809 | 0.00516663 | Serine/Threonine protein kinases, catalytic domain |
| sy134TRINITY_DN3288_c0_g1_i4.p2 |  | 0.000024338 | 0.001801012 | zinc finger |
| sy134TRINITY_DN3850_c0_g1_i1.p1 | Rab7 | 0.000354831 | 0.023064015 | RAB7A, member RAS oncogene family |
| sy134TRINITY_DN7946_c0_g1_i5.p2 | PIF1 | 0.000085981 | 0.005932689 | DNA-dependent ATPase and 5'-3' DNA helicase required for the  maintenance of both mitochondrial and nuclear genome stability |
| xishaHK74SY85_g15022 | OTOA | 0.000000001 | 8.00E-08 | cell-matrix adhesion |
| xishaHK74SY85_g2831 | EPHX2 | 0.000000208 | 1.64E-05 | Epoxide hydrolase 2 |
| nemantTRINITY_DN525_c0_g1_i12.p1 | IK | 0 | 0 | protein localization to kinetochore |
| diadumTRINITY_DN25022_c0_g1_i3.p1 | ATAD2B | 0 | 0 | bromo domain |
| exaipTRINITY_DN21461_c0_g1_i2.p1 |  | 0.000213896 | 0.011122592 | carbohydrate binding |
| exaipTRINITY_DN26438_c0_g1_i1.p1 | SLC18B1 | 0.000256173 | 0.013064823 | transmembrane transport |
| exaipTRINITY_DN51282_c0_g1_i1.p1 |  | 0.000156803 | 0.008467362 | WD domain, G-beta repeat |
| metriTRINITY_DN1032_c0_g1_i1.p1 | MPP6 | 0.000009952 | 0.000577216 | Belongs to the MAGUK family |
| metriTRINITY_DN1091_c0_g1_i6.p1 | TLL1 | 0.000349522 | 0.0174761 | tolloid-like |
| metriTRINITY_DN2396_c0_g1_i2.p1 |  | 0.000090195 | 0.005141115 | cellular response to calcium ion |
| sy134TRINITY_DN21342_c0_g1_i1.p1 | PAQR3 | 0.000003926 | 0.000231634 | protein localization to Golgi apparatus |
| sy134TRINITY_DN238_c0_g1_i1.p1 | PRDM9 | 0.000105385 | 0.00590156 | recombination hotspot binding |
| sy134TRINITY_DN317_c0_g1_i6.p1 | TNIP1 | 0.000000015 | 9.15E-07 | modulation by symbiont of host I-kappaB kinase/NF-kappaB  cascade |
| sy134TRINITY_DN58_c0_g1_i4.p1 | YY1 | 0.000461385 | 0.022607865 | response to prostaglandin F |
| xishaHK74SY85_g15545 | COQ6 | 0.000512782 | 0.024613536 | FAD-dependent monooxygenase required for the C5-ring  hydroxylation during ubiquinone biosynthesis. Catalyzes the  hydroxylation of 3-polyprenyl-4-hydroxybenzoic acid to  3- polyprenyl-4,5-dihydroxybenzoic acid. The electrons required  for the hydroxylation reaction may be funneled indirectly from  NADPH via a ferredoxin ferredoxin reductase system to COQ6 |
| xishaHK74SY85_g20968 |  | 0 | 0 | thiol-dependent ubiquitin-specific protease activity |
| exaipTRINITY_DN24393_c0_g1_i3.p1 | UCKL1 | 0.000779887 | 0.038214463 | uridine kinase activity |
| metriTRINITY_DN16_c0_g2_i1.p1 |  | 0.00000007 | 4.34E-06 |  |
| metriTRINITY_DN2165_c0_g1_i1.p2 | TMX1 | 0.000124168 | 0.006580904 | protein disulfide isomerase activity |
| metriTRINITY_DN2601_c0_g1_i1.p1 | Iap2 | 0.000037105 | 0.002189195 | inhibition of cysteine-type endopeptidase activity involved in  apoptotic process |

**Table S3.** List of positively selected genes from vent sea anemone as fore branch. Ortholog, Gene symbol, p-value, adjusted p-value, Annotation is listed-Continued

| ortholog | gene symbol | p-value | adjusted p-value | annotation |
| --- | --- | --- | --- | --- |
| metriTRINITY_DN6188_c0_g3_i2.p1 |  | 0.000028515 | 0.0017109 | Transferase activity, transferring acyl groups other than amino acyl  groups |
| metriTRINITY_DN805_c0_g1_i1.p1 | ERP44 | 0.000930345 | 0.043726215 | endoplasmic reticulum |
| nemantTRINITY_DN2792_c0_g1_i23.p1 | LRRC41 | 0.000856092 | 0.041092416 | Leucine-rich repeat-containing protein 41 |
| sy134TRINITY_DN7472_c1_g1_i1.p1 | RNGTT | 0.000050305 | 0.00291769 | triphosphatase activity |
| xishaHK74SY85_g16207 |  | 0.000174862 | 0.009092824 |  |
| xishaHK74SY85_g6681 | PLSCR1 | 0.00037157 | 0.01895007 | phospholipid scramblase activity |
| xishaHK74SY85_g7391 | PPIL3 | 0.000070742 | 0.00389081 | peptidyl-prolyl cis-trans isomerase activity |
| diadumTRINITY_DN24360_c0_g1_i1.p1 | SEC62 | 0.000086319 | 0.004661226 | posttranslational protein targeting to endoplasmic reticulum  membrane |
| exaipTRINITY_DN48195_c0_g1_i1.p1 | CHMP7 | 0.000657359 | 0.03286795 | late endosome to vacuole transport |
| sy134TRINITY_DN2406_c0_g1_i2.p1 | UNC13B | 0 | 0 | unc-13 homolog B |
| callic64872_g1_i1.p1 | CSTF1 | 0 | 0 | mRNA processing |
| diadumTRINITY_DN17202_c0_g1_i1.p1 |  | 0.000195484 | 0.009383232 |  |
| diadumTRINITY_DN18286_c1_g1_i1.p1 |  | 0.000905532 | 0.037126812 |  |
| diadumTRINITY_DN21225_c1_g1_i1.p1 | GTF2B | 0.000090095 | 0.00468494 | DNA-templated transcriptional preinitiation complex assembly |
| diadumTRINITY_DN22322_c0_g2_i4.p1 | MTMR9 | 0.000025156 | 0.00138358 | Belongs to the protein-tyrosine phosphatase family. Non-receptor  class myotubularin subfamily |
| diadumTRINITY_DN25965_c1_g1_i4.p1 |  | 0.000224433 | 0.010548351 | metal ion binding |
| exaipTRINITY_DN11822_c0_g2_i1.p1 | METTL17 | 0.000628707 | 0.027034401 | Methyltransferase-like protein 17 |
| exaipTRINITY_DN25913_c0_g1_i1.p1 |  | 0.000007354 | 0.00044124 | calcium ion transmembrane transport |
| exaipTRINITY_DN27180_c0_g1_i3.p1 | ADSL | 0.000127188 | 0.006232212 | (S)-2-(5-amino-1-(5-phospho-D-ribosyl) imidazole-4-carboxamido)  succinate AMP-lyase (fumarate-forming) activity |
| metriTRINITY_DN7632_c2_g1_i1.p1 |  | 0.000013818 | 0.000801444 |  |
| nemantTRINITY_DN10041_c0_g1_i11.p1 | ABT1 | 0.000002249 | 0.000137189 | small subunit processome assembly |
| sy134TRINITY_DN11828_c0_g2_i1.p1 |  | 0.000426721 | 0.019629166 | metallocarboxypeptidase activity |
| sy134TRINITY_DN2250_c0_g2_i1.p1 | AP5B1 | 0.000027333 | 0.001475982 | endosomal transport |
| xishaHK74SY85_g13632 | CFAP20 | 0.000036749 | 0.001947697 | UPF0468 protein C16orf80 homolog |
| xishaHK74SY85_g28316 | SPICE1 | 0.000110603 | 0.00553015 | spindle and centriole associated protein 1 |
| diadumTRINITY_DN28186_c0_g1_i4.p1 | CXXC1 | 0.000474856 | 0.02136852 | unmethylated CpG binding |
| metriTRINITY_DN4532_c0_g1_i1.p1 |  | 0.000016522 | 0.000925232 |  |
| callic63515_g1_i1.p1 | SIRT6 | 0.000030616 | 0.002326816 | NAD-dependent protein deacetylase sirtuin-6 |
| metriTRINITY_DN2175_c0_g1_i1.p1 | TTC25 | 0.000109315 | 0.00787068 | Tetratricopeptide repeats |
| nemantTRINITY_DN3486_c0_g1_i6.p2 | C17orf49 | 0.000159772 | 0.011024268 | chromatin organization |
| nemantTRINITY_DN5406_c0_g1_i2.p1 |  | 0.000000487 | 4.04E-05 | cobalamin catabolic process |
| nemantTRINITY_DN7874_c0_g1_i1.p1 |  | 0.000209868 | 0.014061156 | phosphatidylserine exposure on apoptotic cell surface |
| sy134TRINITY_DN1271_c7_g1_i2.p1 | FLOT1 | 0.000001147 | 9.18E-05 | positive regulation of cell-cell adhesion mediated by cadherin |
| sy134TRINITY_DN3961_c0_g2_i1.p1 |  | 0.000186077 | 0.012653236 | Metal ion binding |
| sy134TRINITY_DN683_c7_g1_i1.p1 | SFT2D2 | 0.000624296 | 0.039954944 | vesicle-mediated transport |
| metriTRINITY_DN1471_c0_g1_i3.p1 |  | 0.000107056 | 0.007815088 | Cupin-like domain |

**Table S3.** List of positively selected genes from vent sea anemone as fore branch. Ortholog, Gene symbol, p-value, adjusted p-value, Annotation is listed-Continued

| ortholog | gene symbol | p-value | adjusted p-value | annotation |
| --- | --- | --- | --- | --- |
| metriTRINITY_DN9550_c0_g3_i1.p2 | C1orf158 | 0.000471837 | 0.031141242 | protein C1orf158 homolog |
| xishaHK74SY85_g23996 | EPS15 | 0.000018817 | 0.001448909 | epidermal growth factor receptor |
| callic45110_g1_i2.p1 |  | 0.000055244 | 0.003259396 | Sel1-like repeats. |
| callic62768_g1_i4.p1 | PARK2 | 0.000012151 | 0.000753362 | regulation of intralumenal vesicle formation |
| diadumTRINITY_DN13589_c0_g2_i2.p1 |  | 0.000774946 | 0.043396976 | [heparan sulfate]-glucosamine 3-sulfotransferase 1 activity |
| diadumTRINITY_DN25621_c0_g1_i1.p1 | SLC4A1AP | 0.000150208 | 0.008712064 | Solute carrier family 4 (anion exchanger), member 1, adaptor  protein |
| sy134TRINITY_DN2546_c1_g1_i1.p2 | WDR81 | 0 | 0 | WD repeat-containing protein 81 |
| sy134TRINITY_DN4874_c3_g1_i11.p3 |  | 0.00000652 | 0.0004238 | Ankyrin repeats (many copies) |
| xishaHK74SY85_g9578 |  | 0.000009337 | 0.000588231 | Galactose-3-O-sulfotransferase |
| metriTRINITY_DN7112_c3_g1_i1.p1 | CDC23 | 0.000002266 | 0.000149556 | regulation of mitotic metaphase/anaphase transition |
| sy134TRINITY_DN18654_c0_g1_i6.p1 |  | 0.000000008 | 5.44E-07 | BTB/POZ domain |
| sy134TRINITY_DN20729_c0_g1_i1.p1 | JPH3 | 0.000550448 | 0.031375536 | regulation of ryanodine-sensitive calcium-release channel activity |
| metriTRINITY_DN9550_c0_g3_i1.p2 | C1orf158 | 0.000471837 | 0.031141242 | protein C1orf158 homolog |
| xishaHK74SY85_g23996 | EPS15 | 0.000018817 | 0.001448909 | epidermal growth factor receptor |
| callic45110_g1_i2.p1 |  | 0.000055244 | 0.003259396 | Sel1-like repeats. |
| callic62768_g1_i4.p1 | PARK2 | 0.000012151 | 0.000753362 | regulation of intralumenal vesicle formation |
| diadumTRINITY_DN13589_c0_g2_i2.p1 |  | 0.000774946 | 0.043396976 | [heparan sulfate]-glucosamine 3-sulfotransferase 1 activity |
| diadumTRINITY_DN25621_c0_g1_i1.p1 | SLC4A1AP | 0.000150208 | 0.008712064 | Solute carrier family 4 (anion exchanger), member 1, adaptor  protein |
| sy134TRINITY_DN2546_c1_g1_i1.p2 | WDR81 | 0 | 0 | WD repeat-containing protein 81 |
| sy134TRINITY_DN4874_c3_g1_i11.p3 |  | 0.00000652 | 0.0004238 | Ankyrin repeats (many copies) |
| xishaHK74SY85_g9578 |  | 0.000009337 | 0.000588231 | Galactose-3-O-sulfotransferase |
| metriTRINITY_DN7112_c3_g1_i1.p1 | CDC23 | 0.000002266 | 0.000149556 | regulation of mitotic metaphase/anaphase transition |
| sy134TRINITY_DN18654_c0_g1_i6.p1 |  | 0.000000008 | 5.44E-07 | BTB/POZ domain |
| sy134TRINITY_DN20729_c0_g1_i1.p1 | JPH3 | 0.000550448 | 0.031375536 | regulation of ryanodine-sensitive calcium-release channel activity |
| metriTRINITY_DN9550_c0_g3_i1.p2 | C1orf158 | 0.000471837 | 0.031141242 | protein C1orf158 homolog |
| xishaHK74SY85_g23996 | EPS15 | 0.000018817 | 0.001448909 | epidermal growth factor receptor |
| callic45110_g1_i2.p1 |  | 0.000055244 | 0.003259396 | Sel1-like repeats. |
| callic62768_g1_i4.p1 | PARK2 | 0.000012151 | 0.000753362 | regulation of intralumenal vesicle formation |
| diadumTRINITY_DN13589_c0_g2_i2.p1 |  | 0.000774946 | 0.043396976 | [heparan sulfate]-glucosamine 3-sulfotransferase 1 activity |
| diadumTRINITY_DN25621_c0_g1_i1.p1 | SLC4A1AP | 0.000150208 | 0.008712064 | Solute carrier family 4 (anion exchanger), member 1, adaptor  protein |
| sy134TRINITY_DN2546_c1_g1_i1.p2 | WDR81 | 0 | 0 | WD repeat-containing protein 81 |
| sy134TRINITY_DN4874_c3_g1_i11.p3 |  | 0.00000652 | 0.0004238 | Ankyrin repeats (many copies) |
| xishaHK74SY85_g9578 |  | 0.000009337 | 0.000588231 | Galactose-3-O-sulfotransferase |
| metriTRINITY_DN7112_c3_g1_i1.p1 | CDC23 | 0.000002266 | 0.000149556 | regulation of mitotic metaphase/anaphase transition |
| sy134TRINITY_DN18654_c0_g1_i6.p1 |  | 0.000000008 | 5.44E-07 | BTB/POZ domain |
| sy134TRINITY_DN20729_c0_g1_i1.p1 | JPH3 | 0.000550448 | 0.031375536 | regulation of ryanodine-sensitive calcium-release channel activity |

**Table S4. List of positively selected genes with both vent and deep-sea anemone as fore branch.** Ortholog, Gene symbol, p-value, adjusted p-value, Annotation is listed.

| Ortholog | Gene symbol | P-value | Adjusted p-value | Annotation |
| --- | --- | --- | --- | --- |
| diadumTRINITY_DN26332_c0_g1_i1.p1 | TIMMDC1 | 0.000000004 | 3.24E-07 | Tim17/Tim22/Tim23/Pmp24 family |
| exaipTRINITY_DN20209_c0_g1_i1.p2 | PRPF18 | 0.000004018 | 0.000317422 | factor 18 |
| metriTRINITY_DN6950_c0_g1_i2.p1 | DAGLB | 0.000394472 | 0.028401984 | retrograde trans-synaptic signaling by lipid |
| metriTRINITY_DN7055_c0_g1_i4.p1 | ARID3A | 0.000000036 | 2.88E-06 | positive regulation of transcription by RNA polymerase II |
| sy134TRINITY_DN1171_c1_g2_i2.p1 | DCHS2 | 0.0006305 | 0.0447655 | condensed mesenchymal cell proliferation |
| sy134TRINITY_DN29_c0_g2_i2.p1 | TRNAU1AP | 0 | 0 | tRNA selenocysteine |
| sy134TRINITY_DN748_c4_g3_i1.p1 | LIN9 | 0.000047993 | 0.003599475 | Lin-9 DREAM MuvB core complex component |
| xishaHK74SY85_g27083 | ATP9B | 0.000009622 | 0.000740894 | ATPase, class II, type 9B |
| diadumTRINITY_DN23065_c0_g1_i1.p1 |  | 0 | 0 |  |
| diadumTRINITY_DN25944_c1_g1_i1.p1 | LIG1 | 0.000331988 | 0.030874884 | Okazaki fragment processing involved in mitotic DNA replication |
| exaipTRINITY_DN26671_c0_g1_i1.p1 |  | 0.00054154 | 0.04819706 | intracellular chloride channel activity |
| exaipTRINITY_DN90456_c0_g1_i1.p1 |  | 0.000043528 | 0.004396328 |  |
| metriTRINITY_DN15415_c0_g1_i1.p1 |  | 0.000039888 | 0.004068576 | Vacuolar Protein |
| metriTRINITY_DN31355_c0_g1_i1.p1 |  | 0.00008925 | 0.0087465 | WD domain, G-beta repeat |
| metriTRINITY_DN435_c1_g4_i1.p1 | SLC25A42 | 0 | 0 | coenzyme A transmembrane transporter activity |
| metriTRINITY_DN4697_c1_g1_i1.p1 | UBE2G1 | 0.000044418 | 0.0044418 | protein modification by small protein conjugation |
| metriTRINITY_DN50_c3_g4_i1.p1 | SLC25A38 | 0.000332041 | 0.030874884 | glycine imports into mitochondrion |
| sy134TRINITY_DN11500_c0_g1_i6.p1 |  | 0 | 0 | glutaminyl-peptide cyclotransferase activity |
| sy134TRINITY_DN1708_c5_g1_i1.p1 | TMUB2 | 0.00007088 | 0.00701712 | transmembrane and ubiquitin-like |
| xishaHK74SY85_g15022 | OTOA | 0.000000027 | 2.94E-06 | cell-matrix adhesion |
| xishaHK74SY85_g2298 | nid2 | 0.000033059 | 0.003438136 | Nidogen 2a (osteonidogen) |
| xishaHK74SY85_g6127 | rap-1 | 0.000528336 | 0.04755024 | Rap protein signal transduction |
| exaipTRINITY_DN11463_c0_g1_i1.p1 |  | 0.000002927 | 0.000313189 | Promotes mitochondrial protein synthesis. May act as a fidelity  factor of the translation reaction, by catalyzing a one- codon  backward translocation of tRNAs on improperly translocated  ribosomes. Binds to mitochondrial ribosomes in a GTP-dependent  manner |
| metriTRINITY_DN28574_c0_g1_i1.p1 |  | 0.000221076 | 0.020781144 |  |
| sy134TRINITY_DN18973_c0_g2_i19.p1 | TBATA | 0.000000897 | 9.69E-05 | Thymus, brain and testes associated |
| diadumTRINITY_DN18735_c0_g2_i4.p1 | RAB3IL1 | 0.000326325 | 0.0274113 | Rab guanyl-nucleotide exchange factor activity |
| diadumTRINITY_DN25309_c0_g1_i3.p1 | MORN3 | 0.000000069 | 6.69E-06 | MORN repeat containing 3 |
| exaipTRINITY_DN19584_c0_g1_i5.p1 | TLL1 | 0 | 0 | tolloid-like |
| exaipTRINITY_DN27278_c1_g1_i1.p1 | RRS1 | 0.000125493 | 0.011168877 | involved in ribosomal large subunit assembly |
| metriTRINITY_DN5883_c0_g1_i1.p1 | RAB1A | 0.000056001 | 0.005096091 | GTPase activity |
| metriTRINITY_DN9596_c1_g1_i3.p1 |  | 0.00001275 | 0.001173 |  |
| nemantTRINITY_DN1582_c1_g1_i6.p1 | KTI12 | 0.000197355 | 0.01736724 | ATP binding |
| xishaHK74SY85_g20063 | ANKRD7 | 0.000263418 | 0.022653948 | negative regulation of fat cell differentiation |
| diadumTRINITY_DN27012_c0_g1_i10.p1 | C11orf16 | 0 | 0 | Domain of unknown function (DUF4537) |
| metriTRINITY_DN3336_c0_g1_i1.p1 | Wnt6 | 0.000011093 | 0.001042742 | Ligand for members of the frizzled family of seven transmembrane  receptors |

**Table S4. List of positively selected genes with both vent and deep-sea anemone as fore branch.** Ortholog, Gene symbol, p-value, adjusted p-value, Annotation is listed-Continued.

| Ortholog | Gene symbol | P-value | Adjusted p-value | Annotation |
| --- | --- | --- | --- | --- |
| metriTRINITY_DN869_c0_g1_i2.p1 | C4orf51 | 0.000077472 | 0.00697248 | protein C4orf51 homolog |
| sy134TRINITY_DN795_c0_g2_i2.p1 | PPP1R12A | 0 | 0 | Protein phosphatase 1 regulatory |
| callic59957_g1_i1.p1 | RRP8 | 0.00040413 | 0.0363717 | Ribosomal RNA processing 8, methyltransferase, homolog (yeast) |
| diadumTRINITY_DN23633_c0_g1_i3.p1 | PMS1 | 0.000123551 | 0.011490243 | DNA mismatch repair protein, C-terminal domain |
| diadumTRINITY_DN24360_c0_g1_i1.p1 | SEC62 | 0.000039649 | 0.003806304 | posttranslational protein targeting to endoplasmic reticulum  membrane |
| metriTRINITY_DN11554_c1_g3_i1.p1 | RBPJ | 0.000020134 | 0.001973132 | positive regulation of canonical Wnt signaling pathway involved in  cardiac muscle cell fate commitment |
| metriTRINITY_DN16_c0_g2_i1.p1 |  | 0.000158377 | 0.014570684 |  |
| metriTRINITY_DN35257_c0_g1_i1.p1 | CDK5 | 0 | 0 | belongs to the protein kinase superfamily |
| metriTRINITY_DN3967_c0_g1_i2.p1 |  | 0.000000436 | 4.49E-05 | coiled coil and C2 |
| metriTRINITY_DN4957_c0_g1_i2.p1 | TPGS2 | 0.000054863 | 0.005157122 | tubulin polyglutamylase complex subunit |
| sy134TRINITY_DN6478_c0_g1_i15.p1 | NOL9 | 0.0000004 | 4.16E-05 | polynucleotide 5'-hydroxyl-kinase activity |
| xishaHK74SY85_g1868 | FBXO8 | 0.000000017 | 1.80E-06 | regulation of ARF protein signal transduction |
| diadumTRINITY_DN28702_c0_g1_i3.p1 |  | 0.000021601 | 0.002095297 | establishment or maintenance of actin cytoskeleton polarity |
| diadumTRINITY_DN25404_c0_g1_i3.p1 | TTC1 | 0.000000287 | 3.24E-05 | Tetratricopeptide repeat |
| exaipTRINITY_DN22683_c0_g1_i1.p1 | PNN | 0.000004849 | 0.000515419 | Pinin, desmosome associated protein |
| metriTRINITY_DN1456_c0_g2_i1.p1 | RTKN | 0.000046039 | 0.004649939 | Rhotekin |
| metriTRINITY_DN1914_c2_g3_i1.p1 | RAB14 | 0.00000152 | 0.00016568 | Golgi to endosome transport |
| metriTRINITY_DN2664_c1_g1_i1.p1 | DDR2 | 0.000111303 | 0.0111303 | Discoidin domain-containing receptor 2-like |
| metriTRINITY_DN3524_c0_g1_i3.p1 | VWA8 | 0.000000003 | 3.57E-07 | ATPase activity |
| metriTRINITY_DN3719_c2_g2_i1.p1 |  | 0.000044089 | 0.004497078 | distal tubule morphogenesis |
| metriTRINITY_DN4532_c0_g1_i1.p1 |  | 0.000001195 | 0.000132645 |  |
| metriTRINITY_DN9913_c0_g1_i1.p1 | SCNM1 | 0.00000007 | 8.05E-06 | RNA splicing |
| nemantTRINITY_DN4192_c1_g1_i12.p1 | AARS | 0.000001161 | 0.000130032 | Catalyzes the attachment of alanine to tRNA(Ala) in a two-step  reaction alanine is first activated by ATP to form Ala- AMP and  then transferred to the acceptor end of tRNA(Ala). Also edits  incorrectly charged tRNA(Ala) via its editing domain |
| nemantTRINITY_DN7306_c0_g1_i4.p1 | ANKRD16 | 0.000160726 | 0.015751148 | Ankyrin repeat |
| nemantTRINITY_DN963_c0_g1_i16.p1 |  | 0.000126804 | 0.012553596 | macromolecule localization |
| xishaHK74SY85_g1031 | ESF1 | 0.000001437 | 0.00015807 | nucleic acid-templated transcription |
| xishaHK74SY85_g13632 | CFAP20 | 0.000038361 | 0.003951183 | UPF0468 protein C16orf80 homolog |
| metriTRINITY_DN34956_c0_g1_i1.p1 |  | 0.000004626 | 0.000499608 | cytochrome P450 |
| sy134TRINITY_DN4614_c0_g1_i1.p1 | CARHSP1 | 0.000430231 | 0.040871945 | mRNA 3'-UTR binding |
| sy134TRINITY_DN6293_c0_g1_i17.p1 | ALS2CR12 | 0.000000007 | 8.26E-07 | Amyotrophic lateral sclerosis 2 |
| xishaHK74SY85_g23376 | STKLD1 | 0.000000007 | 8.26E-07 | Protein tyrosine kinase |
| callic51843_g1_i1.p1 | DPH1 | 0.000114553 | 0.012902754 | peptidyl-diphthamide biosynthetic process from peptidyl-histidine |
| exaipTRINITY_DN27421_c0_g1_i1.p2 | PUS10 | 0.046641616 | 3.52E-06 | tRNA pseudouridine synthesis |
| exaipTRINITY_DN33943_c0_g1_i1.p1 | dhhc-8 | 0.026325373 | 0.003075 | protein-cysteine S-acyltransferase activity |
| metriTRINITY_DN11664_c0_g1_i8.p1 |  | 0.018414714 | 4.89E-05 | GABA-A receptor activity |

**Table S4. List of positively selected genes with both vent and deep-sea anemone as fore branch.** Ortholog, Gene symbol, p-value, adjusted p-value, Annotation is listed-Continued.

| Ortholog | Gene symbol | P-value | Adjusted p-value | Annotation |
| --- | --- | --- | --- | --- |
| metriTRINITY_DN3856_c0_g1_i2.p1 |  | 0.042729041 | 0.030163749 | Calcium-activated chloride channel |
| nemantTRINITY_DN3172_c0_g1_i12.p1 | RNF20 | 0.000386408 | 4.73E-05 | histone monoubiquitination |
| nemantTRINITY_DN9368_c0_g2_i5.p1 | PRKAA1 | 0.000001721 | 0.00065858 | 5'-AMP-activated protein kinase catalytic subunit |
| sy134TRINITY_DN11940_c0_g1_i2.p1 | ATP5C1 | 0.032808392 | 0.020571798 | ATP synthase |
| xishaHK74SY85_g5988 | POC5 | 0.007906196 | 1.87E-06 | cell cycle |
| metriTRINITY_DN1479_c1_g1_i1.p1 | CLIC6 | 0.007303451 | 0.00202146 | chloride channel activity |
| metriTRINITY_DN3037_c0_g1_i2.p2 |  | 0 | 0.023054287 |  |
| metriTRINITY_DN4237_c0_g1_i1.p1 |  | 0 | 0.000000006 | voltage-gated potassium channel activity |
| metriTRINITY_DN5642_c0_g2_i8.p1 |  | 0 | 0.00000077 | 5-methyltetrahydrofolate-dependent methyltransferase activity |
| metriTRINITY_DN7578_c1_g1_i1.p1 | SLC25A34 | 0 | 0 | mitochondrial transport |
| nemantTRINITY_DN7874_c0_g1_i1.p1 |  | 0.000000569 | 0.000000342 | phosphatidylserine exposure on apoptotic cell surface |
| sy134TRINITY_DN1656_c0_g2_i2.p1 | Rab5 | 0.000002344 | 0.001887824 | member RAS oncogene family |
| metriTRINITY_DN3856_c0_g1_i2.p1 |  | 0.042729041 | 0.030163749 | Calcium-activated chloride channel |
| nemantTRINITY_DN3172_c0_g1_i12.p1 | RNF20 | 0.000386408 | 4.73E-05 | histone monoubiquitination |
| nemantTRINITY_DN9368_c0_g2_i5.p1 | PRKAA1 | 0.000001721 | 0.00065858 | 5'-AMP-activated protein kinase catalytic subunit |
| sy134TRINITY_DN11940_c0_g1_i2.p1 | ATP5C1 | 0.032808392 | 0.020571798 | ATP synthase |
| xishaHK74SY85_g5988 | POC5 | 0.007906196 | 1.87E-06 | cell cycle |
| metriTRINITY_DN1479_c1_g1_i1.p1 | CLIC6 | 0.007303451 | 0.00202146 | chloride channel activity |
| metriTRINITY_DN3037_c0_g1_i2.p2 |  | 0 | 0.023054287 |  |
| metriTRINITY_DN4237_c0_g1_i1.p1 |  | 0 | 0.000000006 | voltage-gated potassium channel activity |
| metriTRINITY_DN5642_c0_g2_i8.p1 |  | 0 | 0.00000077 | 5-methyltetrahydrofolate-dependent methyltransferase activity |
| metriTRINITY_DN7578_c1_g1_i1.p1 | SLC25A34 | 0 | 0 | mitochondrial transport |
| nemantTRINITY_DN7874_c0_g1_i1.p1 |  | 0.000000569 | 0.000000342 | phosphatidylserine exposure on apoptotic cell surface |
| sy134TRINITY_DN1656_c0_g2_i2.p1 | Rab5 | 0.000002344 | 0.001887824 | member RAS oncogene family |
| metriTRINITY_DN3856_c0_g1_i2.p1 |  | 0.042729041 | 0.030163749 | Calcium-activated chloride channel |
| nemantTRINITY_DN3172_c0_g1_i12.p1 | RNF20 | 0.000386408 | 4.73E-05 | histone monoubiquitination |
| nemantTRINITY_DN9368_c0_g2_i5.p1 | PRKAA1 | 0.000001721 | 0.00065858 | 5'-AMP-activated protein kinase catalytic subunit |
| sy134TRINITY_DN11940_c0_g1_i2.p1 | ATP5C1 | 0.032808392 | 0.020571798 | ATP synthase |
| xishaHK74SY85_g5988 | POC5 | 0.007906196 | 1.87E-06 | cell cycle |
| metriTRINITY_DN1479_c1_g1_i1.p1 | CLIC6 | 0.007303451 | 0.00202146 | chloride channel activity |
| metriTRINITY_DN3037_c0_g1_i2.p2 |  | 0 | 0.023054287 |  |
| metriTRINITY_DN4237_c0_g1_i1.p1 |  | 0 | 0.000000006 | voltage-gated potassium channel activity |
| metriTRINITY_DN5642_c0_g2_i8.p1 |  | 0 | 0.00000077 | 5-methyltetrahydrofolate-dependent methyltransferase activity |
| metriTRINITY_DN7578_c1_g1_i1.p1 | SLC25A34 | 0 | 0 | mitochondrial transport |
| nemantTRINITY_DN7874_c0_g1_i1.p1 |  | 0.000000569 | 0.000000342 | phosphatidylserine exposure on apoptotic cell surface |
| sy134TRINITY_DN1656_c0_g2_i2.p1 | Rab5 | 0.000002344 | 0.001887824 | member RAS oncogene family |
| xishaHK74SY85_g10333 |  | 0.000007748 | 0.000009479 | gamma-aminobutyric acid:proton symporter activity |
| xishaHK74SY85_g11169 | SCYL3 | 0.0000332 | 0.000044423 | ATP binding |
| xishaHK74SY85_g25407 |  | 0.0000609 | 0.000269428 | alpha1-adrenergic receptor activity |
| xishaHK74SY85_g5847 | RHOT2 | 0.0000517 | 0.002236691 | Mitochondrial GTPase involved in mitochondrial trafficking |

**Table S4. List of positively selected genes with both vent and deep-sea anemone as fore branch.** Ortholog, Gene symbol, p-value, adjusted p-value, Annotation is listed-Continued.

| Ortholog | Gene symbol | P-value | Adjusted p-value | Annotation |
| --- | --- | --- | --- | --- |
| diadumTRINITY_DN24773_c0_g1_i3.p1 | BTRC | 0.000592148 | 0.044094975 | SCF-dependent proteasomal ubiquitin-dependent protein catabolic  process |
| diadumTRINITY_DN27714_c0_g1_i1.p1 | YTHDC1 | 0.000032799 | 0.002722317 | YTH domain containing 1 |
| exaipTRINITY_DN23832_c0_g1_i3.p1 | TGFB1I1 | 0.00000004 | 3.52E-06 | Roundabout binding |
| exaipTRINITY_DN24554_c1_g2_i1.p1 | RTCB | 0.0000375 | 0.003075 | RNA ligase (ATP) activity |
| exaipTRINITY_DN6853_c0_g1_i1.p1 | ALG14 | 0.000000569 | 4.89E-05 | dolichol-linked oligosaccharide biosynthetic process |
| metriTRINITY_DN467_c0_g1_i2.p1 | SMNDC1 | 0.000041584 | 0.003368304 | RNA splicing |
| metriTRINITY_DN6783_c4_g1_i1.p1 |  | 0.000000544 | 4.73E-05 | Ion transport protein |
| metriTRINITY_DN8341_c0_g1_i2.p1 |  | 0.000007748 | 0.00065858 | calcium ion-regulated exocytosis of neurotransmitter |
| metriTRINITY_DN90_c3_g2_i2.p2 | SGPL1 | 0.000263741 | 0.020571798 | sphinganine-1-phosphate aldolase activity |
| exaipTRINITY_DN26944_c0_g1_i1.p1 |  | 0.000631755 | 0.046118115 |  |
| callic64610_g1_i3.p1 | STX16 | 0.000347139 | 0.036449595 | Glycosyltransferase like family |
| exaipTRINITY_DN12612_c0_g2_i1.p1 |  | 0.000000029 | 3.39E-06 | structural molecule activity |
| metriTRINITY_DN1031_c0_g1_i1.p1 |  | 0.000051461 | 0.005684532 |  |
| metriTRINITY_DN11693_c0_g1_i1.p1 | ube2c | 0.000087468 | 0.009534012 | ubiquitin-conjugating enzyme |
| metriTRINITY_DN1770_c0_g4_i1.p1 | LDLRAP1 | 0.000051212 | 0.005684532 | Low density lipoprotein receptor |
| metriTRINITY_DN1998_c0_g1_i8.p1 | ATXN7L3 | 0.000031842 | 0.003598146 | Component of the transcription regulatory histone acetylation  (HAT) complex SAGA, a multiprotein complex that activates  transcription by remodeling chromatin and mediating  histone acetylation and deubiquitination. Within the SAGA  complex, participates in a subcomplex that specifically  deubiquitinates |
| metriTRINITY_DN22191_c0_g1_i1.p1 | cox1 | 0 | 0 | Cytochrome C and Quinol oxidase polypeptide I |
| metriTRINITY_DN4074_c0_g2_i1.p1 | fwd | 0.000001083 | 0.000124545 | Belongs to the PI3 PI4-kinase family |
| metriTRINITY_DN5702_c0_g1_i4.p1 | TRAK2 | 0.000258547 | 0.027664529 | anterograde dendritic transport of mitochondrion |
| metriTRINITY_DN6982_c1_g1_i1.p1 | CCDC186 | 0 | 0 | Rab GTPase binding |
| xishaHK74SY85_g1069 |  | 0 | 0 | Tyrosine kinase, catalytic domain |
| xishaHK74SY85_g3162 | PPP3CA | 0.000104767 | 0.011314836 | calmodulin-dependent protein phosphatase activity |
| exaipTRINITY_DN13638_c0_g1_i1.p1 | EIF2S1 | 0.000005243 | 0.000597702 | translation initiation factor activity |
| exaipTRINITY_DN27414_c1_g1_i1.p1 | TAOK1 | 0.000036137 | 0.004047344 | activation of MAPKK activity |
| exaipTRINITY_DN27853_c1_g1_i2.p1 | TDRKH | 0.000000023 | 2.71E-06 | DNA methylation involved in gamete generation |
| diadumTRINITY_DN24773_c0_g1_i3.p1 | BTRC | 0.000592148 | 0.044094975 | SCF-dependent proteasomal ubiquitin-dependent protein catabolic  process |
| diadumTRINITY_DN27714_c0_g1_i1.p1 | YTHDC1 | 0.000032799 | 0.002722317 | YTH domain containing 1 |
| exaipTRINITY_DN23832_c0_g1_i3.p1 | TGFB1I1 | 0.00000004 | 3.52E-06 | Roundabout binding |
| exaipTRINITY_DN24554_c1_g2_i1.p1 | RTCB | 0.0000375 | 0.003075 | RNA ligase (ATP) activity |
| exaipTRINITY_DN6853_c0_g1_i1.p1 | ALG14 | 0.000000569 | 4.89E-05 | dolichol-linked oligosaccharide biosynthetic process |
| metriTRINITY_DN467_c0_g1_i2.p1 | SMNDC1 | 0.000041584 | 0.003368304 | RNA splicing |
| metriTRINITY_DN6783_c4_g1_i1.p1 |  | 0.000000544 | 4.73E-05 | Ion transport protein |
| metriTRINITY_DN8341_c0_g1_i2.p1 |  | 0.000007748 | 0.00065858 | calcium ion-regulated exocytosis of neurotransmitter |

**Table S4. List of positively selected genes with both vent and deep-sea anemone as fore branch.** Ortholog, Gene symbol, p-value, adjusted p-value, Annotation is listed-Continued.

| Ortholog | Gene symbol | P-value | Adjusted p-value | Annotation |
| --- | --- | --- | --- | --- |
| metriTRINITY_DN90_c3_g2_i2.p2 | SGPL1 | 0.000263741 | 0.020571798 | sphinganine-1-phosphate aldolase activity |
| exaipTRINITY_DN26944_c0_g1_i1.p1 |  | 0.000631755 | 0.046118115 |  |
| callic64610_g1_i3.p1 | STX16 | 0.000347139 | 0.036449595 | Glycosyltransferase like family |
| exaipTRINITY_DN12612_c0_g2_i1.p1 |  | 0.000000029 | 3.39E-06 | structural molecule activity |
| metriTRINITY_DN1031_c0_g1_i1.p1 |  | 0.000051461 | 0.005684532 |  |
| metriTRINITY_DN11693_c0_g1_i1.p1 | ube2c | 0.000087468 | 0.009534012 | ubiquitin-conjugating enzyme |
| metriTRINITY_DN1770_c0_g4_i1.p1 | LDLRAP1 | 0.000051212 | 0.005684532 | Low density lipoprotein receptor |
| metriTRINITY_DN1998_c0_g1_i8.p1 | ATXN7L3 | 0.000031842 | 0.003598146 | Component of the transcription regulatory histone acetylation  (HAT) complex SAGA, a multiprotein complex that activates  transcription by remodeling chromatin and mediating  histone acetylation and deubiquitination. Within the SAGA  complex, participates in a subcomplex that specifically  deubiquitinates |
| metriTRINITY_DN22191_c0_g1_i1.p1 | cox1 | 0 | 0 | Cytochrome C and Quinol oxidase polypeptide I |
| metriTRINITY_DN4074_c0_g2_i1.p1 | fwd | 0.000001083 | 0.000124545 | Belongs to the PI3 PI4-kinase family |
| metriTRINITY_DN5702_c0_g1_i4.p1 | TRAK2 | 0.000258547 | 0.027664529 | anterograde dendritic transport of mitochondrion |
| metriTRINITY_DN6982_c1_g1_i1.p1 | CCDC186 | 0 | 0 | Rab GTPase binding |
| xishaHK74SY85_g1069 |  | 0 | 0 | Tyrosine kinase, catalytic domain |
| xishaHK74SY85_g3162 | PPP3CA | 0.000104767 | 0.011314836 | calmodulin-dependent protein phosphatase activity |
| exaipTRINITY_DN13638_c0_g1_i1.p1 | EIF2S1 | 0.000005243 | 0.000597702 | translation initiation factor activity |
| exaipTRINITY_DN27414_c1_g1_i1.p1 | TAOK1 | 0.000036137 | 0.004047344 | activation of MAPKK activity |
| exaipTRINITY_DN27853_c1_g1_i2.p1 | TDRKH | 0.000000023 | 2.71E-06 | DNA methylation involved in gamete generation |
| sy134TRINITY_DN4222_c0_g1_i11.p1 | MITF | 0.00000022 | 2.55E-05 | canonical Wnt signaling pathway involved in negative regulation of  apoptotic process |
| xishaHK74SY85_g27693 |  | 0.00045703 | 0.04753112 | microtubule motor activity |
| callic50338_g1_i1.p1 | PSMD14 | 0.000194416 | 0.021969008 | Lys63-specific deubiquitinase activity |
| callic58550_g1_i1.p1 | BRD2 | 0.000066835 | 0.00788653 | Bromodomain extra-terminal - transcription regulation |
| diadumTRINITY_DN17361_c0_g1_i1.p1 | KIAA0226 | 0.000251048 | 0.027866328 | RUN and cysteine rich domain containing beclin 1 interacting  protein |
| exaipTRINITY_DN17963_c0_g3_i1.p1 | SMARCA4 | 0.000431477 | 0.047030993 | SWI SNF related, matrix associated, actin dependent regulator of  chromatin, subfamily a, member |
| exaipTRINITY_DN19925_c0_g1_i1.p1 |  | 0.000134069 | 0.015417935 | Belongs to the short-chain dehydrogenases reductases (SDR)  family |
| metriTRINITY_DN16663_c1_g3_i5.p1 |  | 0.000020078 | 0.00240936 | calcium ion binding |
| metriTRINITY_DN2316_c0_g1_i1.p1 |  | 0.000106124 | 0.012310384 | cerebellar neuron development |
| metriTRINITY_DN8662_c0_g1_i1.p1 | RAD54L | 0.000009818 | 0.001197796 | DNA synthesis involved in double-strand break repair via  homologous recombination |
| sy134TRINITY_DN22_c12_g1_i1.p1 |  | 0.000000002 | 2.56E-07 |  |
| xishaHK74SY85_g27396 | TMED7 | 0.000010357 | 0.001253197 | protein transport |
| exaipTRINITY_DN26167_c0_g1_i3.p1 | TAF1 | 0 | 0 | TAF1 RNA polymerase II, TATA box binding protein (TBP)-  associated factor |
| diadumTRINITY_DN21207_c1_g1_i1.p1 | BCAT1 | 0.000206962 | 0.015108226 | L-isoleucine transaminase activity |
| exaipTRINITY_DN20562_c0_g2_i1.p1 |  | 0.000016675 | 0.0012673 | chitin metabolic process |
| nemantTRINITY_DN16679_c0_g1_i8.p1 | ABCF1 | 0.000007555 | 0.00058929 | translation activator activity |

**Table S5** Annotation of specific expanded gene families in the vent sea anemone *Alvinactis* sp.

| Gene family | Gene Symbol | Species | Annotation |
| --- | --- | --- | --- |
| OG0001483. |  | Metazoa | ubiquitin protein ligase binding |
| OG0000701 |  | Metazoa | transposition, RNA-mediated |
| OG0000242 |  | Metazoa | collagen |
| OG0000079 |  | Metazoa | MATH (Meprin-associated Traf homology) domain containing |
| OG0000035 |  | Eukaryota | zinc ion binding |
| OG0000422 |  | Metazoa | Helicase conserved C-terminal domain |
| OG0000904 |  | Metazoa | regulation of mitotic cell cycle |
| OG0000709. |  | Methylococcales | Wzt C-terminal domain |
| OG0002141 |  | Metazoa | negative regulation of non-canonical Wnt signaling pathway |
| OG0000055 |  | Bilateria | Coagulation factor 5/8 C-terminal domain, discoidin domain |
| OG0000797 |  | Metazoa | regulation of mitotic cell cycle |
| OG0002851 | ACSF2 | Eukaryota | o-succinylbenzoate-CoA ligase activity |
| OG0000018 |  | Metazoa | zinc ion binding |
| OG0000574 |  | Eukaryota | protein ubiquitination |
| OG0000871 |  | Bilateria | Fibrinogen-related domains (FReDs) |

**Table S6.** Annotation of shared expanded gene families in vent and deep-sea anemones.

| Gene family | Gene Symbol | Species | Annotation |  |  |  |
| --- | --- | --- | --- | --- | --- | --- |
| OG0000029 |  | Metazoa | Pao retrotransposon peptidase |  |  |  |
| OG0001165 |  | Metazoa | Helicase conserved C-terminal domain |  |  |  |
| OG0000743 | EDIL3 | Metazoa | integrin binding |  |  |  |
| OG0001539 |  | Metazoa | double-stranded DNA 5'-3' exodeoxyribonuclease activity |  |  |  |
| OG0001034 |  | Metazoa | AMP-binding enzyme |  |  |  |
| OG0000594 | ATAD5 | Metazoa | negative regulation of intrinsic apoptotic signaling pathway in response to DNA damage by p53 class mediator |  |  |  |
| OG0000125 |  | Bilateria | transposition, RNA-mediated |  |  |  |
| OG0000324 |  | Metazoa | Reverse transcriptase (RNA-dependent DNA polymerase) |  |  |  |
| OG0001548 |  | Bilateria | Protein of unknown function (DUF1759) |  |  |  |

**Table S**7. Functional enrichment of differentially expressed genes in vent sea anemone (*Alvinacti*s sp.).

| #Term | Input number | Background number | P-Value | Corrected P-Value |
| --- | --- | --- | --- | --- |
| Ribosome | 43 | 116 | 2.50E-22 | 2.73E-20 |
| Metabolic pathways | 115 | 908 | 1.87E-20 | 1.02E-18 |
| Oxidative phosphorylation | 21 | 82 | 3.69E-09 | 1.34E-07 |
| Proteasome | 14 | 40 | 6.05E-08 | 1.65E-06 |
| Endocytosis | 21 | 116 | 6.29E-07 | 1.37E-05 |
| Lysosome | 18 | 93 | 1.71E-06 | 3.10E-05 |
| Carbon metabolism | 17 | 96 | 9.32E-06 | 0.000130855 |
| Spliceosome | 18 | 107 | 9.60E-06 | 0.000130855 |
| mRNA surveillance pathway | 13 | 62 | 2.22E-05 | 0.00025826 |
| Biosynthesis of amino acids | 14 | 73 | 2.60E-05 | 0.00025826 |
| Protein processing in endoplasmic reticulum | 17 | 105 | 2.61E-05 | 0.00025826 |
| N-Glycan biosynthesis | 10 | 38 | 3.79E-05 | 0.000344312 |
| Wnt signaling pathway | 12 | 59 | 5.89E-05 | 0.000456646 |
| RNA transport | 18 | 125 | 6.08E-05 | 0.000456646 |
| mTOR signaling pathway | 14 | 80 | 6.28E-05 | 0.000456646 |
| Phagosome | 12 | 62 | 8.95E-05 | 0.00060958 |
| Autophagy - animal | 12 | 79 | 0.000647897 | 0.004154165 |
| Amino sugar and nucleotide sugar metabolism | 8 | 39 | 0.000948787 | 0.005745432 |
| Mitophagy - animal | 7 | 32 | 0.0014209 | 0.008151478 |
| Pyruvate metabolism | 7 | 33 | 0.001657649 | 0.009034187 |
| Citrate cycle (TCA cycle) | 6 | 28 | 0.003382538 | 0.017556984 |
| Valine, leucine and isoleucine degradation | 7 | 40 | 0.004281633 | 0.021213546 |
| Fatty acid degradation | 6 | 31 | 0.005227167 | 0.02374005 |
| Glyoxylate and dicarboxylate metabolism | 6 | 31 | 0.005227167 | 0.02374005 |
| Peroxisome | 9 | 66 | 0.005636117 | 0.02457347 |
| Fructose and mannose metabolism | 5 | 22 | 0.005939744 | 0.024824878 |
| Ribosome biogenesis in eukaryotes | 9 | 67 | 0.006149282 | 0.024824878 |
| beta-Alanine metabolism | 5 | 23 | 0.006973001 | 0.027144896 |
| Basal transcription factors | 6 | 35 | 0.008711446 | 0.032743023 |
| Pentose and glucuronate interconversions | 4 | 16 | 0.01035627 | 0.03762778 |
| Phosphatidylinositol signaling system | 7 | 49 | 0.011227271 | 0.039476535 |
| Lysine degradation | 6 | 38 | 0.012236548 | 0.04041769 |
| Cysteine and methionine metabolism | 6 | 38 | 0.012236548 | 0.04041769 |
| Propanoate metabolism | 5 | 28 | 0.014052024 | 0.045049136 |

**Table S8**. Hyper-metric tree with divergence time counted by MCMCtree

#NEXUS

BEGIN TREES;

UTREE 1 = (((Edwardsiella_carnea: 1.061943, Nematostella_vectensis: 1.061943): 2.124428, Scolanthus_callimorphus: 3.186371): 0.553338, ((Entacmaea_quadricolor: 2.250661, (Stichodactyla_helianthus: 1.172435, (Aulactinia_veratra: 0.846233, (Condylactis_gigantea: 0.612543, (Anthopleura_elegantissima: 0.386238, (Actinia_equina: 0.170133, Actinia_tenebrosa: 0.170133) : 0.216105) : 0.226305) : 0.233690) : 0.326202) : 1.078226) : 1.181813, (((Diadumene_lineata: 1.350815, Metridium_senile: 1.350815) : 0.565202, Exaiptasia_diaphana: 1.916017) : 0.399069, (Alvinactis_sp: 1.488516, (Paraphelliactis_xishaensis: 0.933731, (Calliactis_polypus: 0.541531, Nemanthus_annamensis: 0.541531) : 0.392201) : 0.554784) : 0.826571) : 1.117388) : 0.307235) ;

END;

**Reference**

Baumgarten S, Simakov O, Esherick LY, Liew YJ, Lehnert EM, Michell CT, Li Y, Hambleton EA, Guse A, Oates ME, Gough J, Weis VM, Aranda M, Pringle JR, Voolstra CR. The genome of Aiptasia, a sea anemone model for coral symbiosis. Proc Natl Acad Sci U S A. 2015 Sep 22;112(38):11893-8. doi: 10.1073/pnas.1513318112. Epub 2015 Aug 31.

Dnyansagar R, Zimmermann B, Moran Y, Praher D, Sundberg P, Møller LF, Technau U. Dispersal and speciation: The cross Atlantic relationship of two parasitic cnidarians. Mol Phylogenet Evol. 2018 Sep;126:346-355. doi: 10.1016/j.ympev.2018.04.035.

Feng, Chenguang & Liu, Ruoyu & Xu, Wenjie & Zhou, Yang & Zhu, Chenglong & Liu, Jun & Wu, Baosheng & Li, Yongxin & Qiu, Qiang & He, Shunping & Wang, Wjdcvt & Zhang, Haibin & Kun, Wang. (2021). The genome of a new anemone species (Actiniaria: Hormathiidae) provides insights into deep-sea adaptation. Deep Sea Research Part I: Oceanographic Research Papers. 170. 103492. 10.1016/j.dsr.2021.103492.

Macrander, J., Brugler, M.R. & Daly, M. A RNA-seq approach to identify putative toxins from acrorhagi in aggressive and non-aggressive Anthopleura elegantissima polyps. BMC Genomics 16, 221 (2015). <https://doi.org/10.1186/s12864-015-1417-4>

Putnam NH, Srivastava M, Hellsten U, Dirks B, Chapman J, Salamov A, Terry A, Shapiro H, Lindquist E, Kapitonov VV, Jurka J, Genikhovich G, Grigoriev IV, Lucas SM, Steele RE, Finnerty JR, Technau U, Martindale MQ, Rokhsar DS. Sea anemone genome reveals ancestral eumetazoan gene repertoire and genomic organization. Science. 2007 Jul 6;317(5834):86-94. doi: 10.1126/science.1139158.

Rivera-de-Torre, Esperanza & Martínez-del-Pozo, Alvaro & Garb, Jessica. (2018). Stichodactyla helianthus ' de novo transcriptome assembly: Discovery of a new actinoporin isoform. Toxicon. 150. 10.1016/j.toxicon.2018.05.014.

Stewart ZK, Pavasovic A, Hock DH, Prentis PJ. Transcriptomic investigation of wound healing and regeneration in the cnidarian Calliactis polypus. Sci Rep. 2017 Feb 2;7:41458. doi: 10.1038/srep41458.

Surm JM, Smith HL, Madio B, Undheim EAB, King GF, Hamilton BR, van der Burg CA, Pavasovic A, Prentis PJ. A process of convergent amplification and tissue-specific expression dominates the evolution of toxin and toxin-like genes in sea anemones. Mol Ecol. 2019 May;28(9):2272-2289. doi: 10.1111/mec.15084.

van der Burg CA, Prentis PJ, Surm JM, Pavasovic A. Insights into the innate immunome of actiniarians using a comparative genomic approach. BMC Genomics. 2016 Nov 2;17(1):850. doi: 10.1186/s12864-016-3204-2.

Wilding CS, Fletcher N, Smith EK, Prentis P, Weedall GD, Stewart Z. The genome of the sea anemone Actinia equina (L.): Meiotic toolkit genes and the question of sexual reproduction. Mar Genomics. 2020 Oct;53:100753. doi: 10.1016/j.margen.2020.100753. Epub 2020 Feb 11.
